# Supplementary material for: Susceptibility to misinformation about COVID-19 around the world
Source: R Soc Open Sci. 2020 Oct 14;7(10):201199. doi: 10.1098/rsos.201199 (PMC7657933; doi:10.1098/rsos.201199)
Supplement: Supplementary Information [file rsos201199supp1.docx]

**Supplementary Information for “Susceptibility to misinformation about COVID-19 around the world”**

**
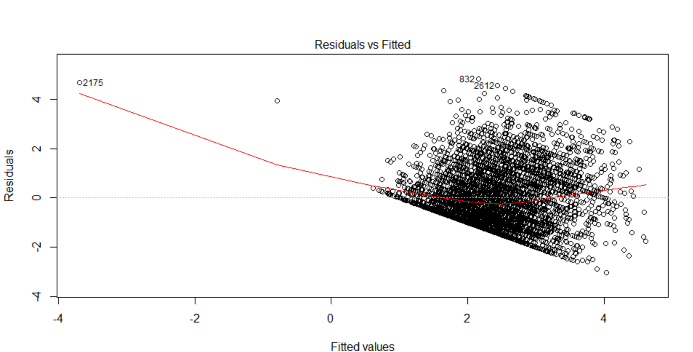

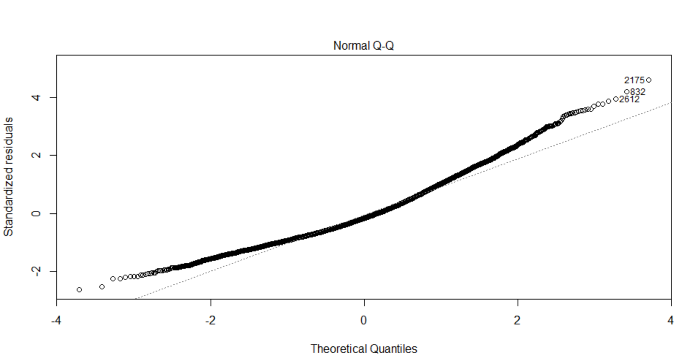
**


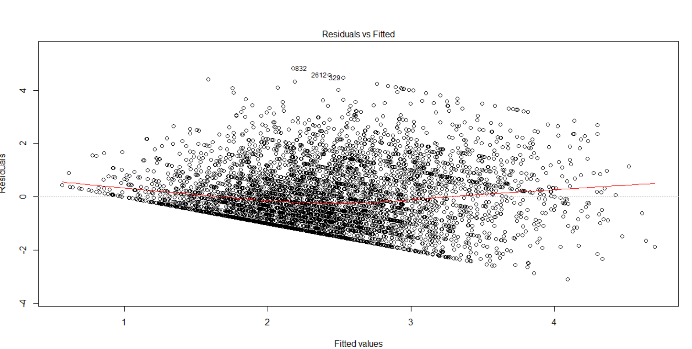

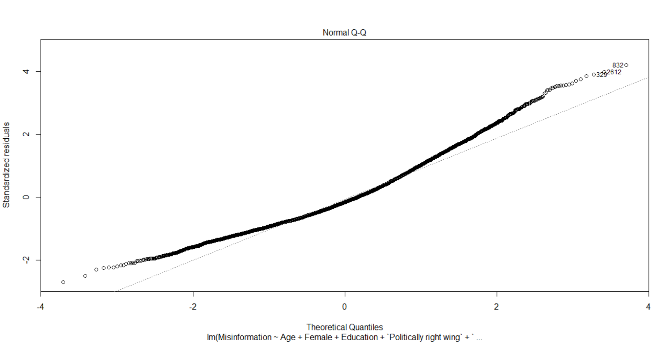


**Figure S1.** Linear regression model diagnostic plots (pooled). *Note:* Top figures represent the residuals plot and quantile-quantile (QQ) plot for the fitted OLS multiple regression model, plotted using R’s plot(model) function. The Durbin-Watson test (1.93, *p* = 0.004, autocorrelation = 0.035) confirmed that the errors were relatively uncorrelated. Two outliers are responsible for the apparent violation of the model’s linearity assumption (e.g. entry 2175); this is due to an error in the text entry for “age”, in which participants indicated to be 699 and 399 years old, respectively. The two bottom figures represent the residuals plot and QQ plot for the fitted model when these two outliers are excluded, indicating improved linearity, and a near-normal distribution of the errors/residuals. We find no meaningful differences between the fitted model with the age outliers left in and when excluded.


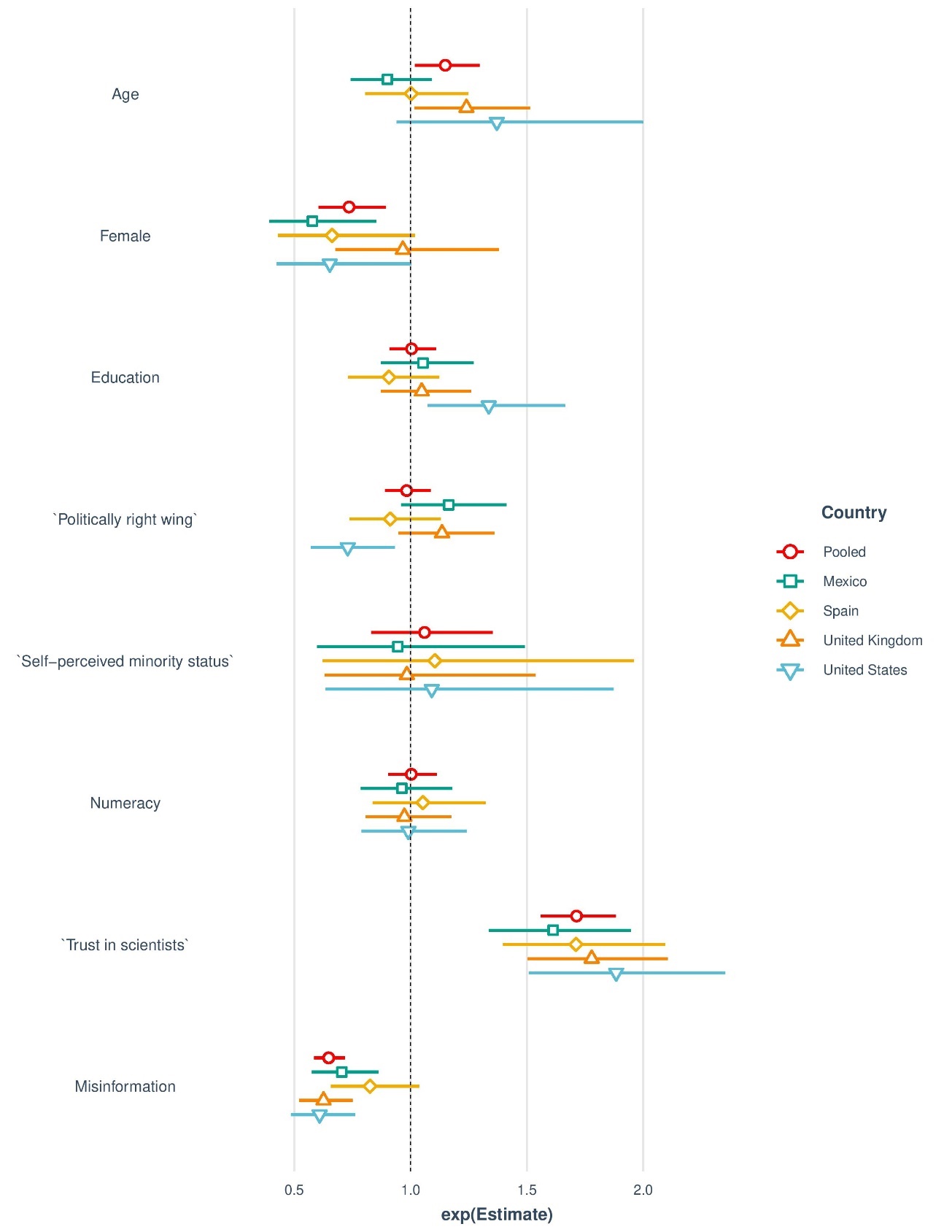


**Figure S2.** Logistic regression model for “Would you recommend getting vaccinated against COVID-19 to vulnerable friends or family members (y/n)”, by country. *Note*: coefficients are exponentiated and represent odds ratios. Left of the dotted line (i.e. negative values) indicate reduced susceptibility to misinformation.

**Table S1.** Misinformation & factual items

| **Item no.** | **Type** | **Item name** | **Full text** |
| --- | --- | --- | --- |
| 1 | Misinformation | Bioengineering | The coronavirus was bioengineered in a military lab in Wuhan. |
| 2 |  | Breath | Being able to hold your breath for 10 seconds or more without coughing or discomfort is a good self-check test for whether you have the coronavirus |
| 3 |  | Vaccination | The coronavirus is part of a global effort to enforce mandatory vaccination. |
| 4 |  | Salt water | Gargling salt water or lemon juice reduces the risk of infection from Coronavirus |
| 5 |  | 5G | The new 5G network may be making us more susceptible to the virus |
| 6 |  | Hot air | Breathing in hot air through your mouth and nose (e.g. from a hair dryer) kills the coronavirus as it can only live in cool places. |
| 7 | Factual information | Diabetes | People with diabetes are at high risk of complications from coronavirus infection |
| 8 |  | Sanitizer | Using hand sanitizer with at least 60% alcohol is effective in reducing risk of infection from coronavirus. |
| 9 | Ambiguous | Ibuprofen | Taking ibuprofen when you are infected with the coronavirus could make your symptoms worse |

**Table S2.** Sample composition

| **Country** | *Pooled* | | *Ireland* | | *Mexico* | | *Spain* | | *UK - April* | | *UK - May* | | *USA* | |
| --- | --- | --- | --- | --- | --- | --- | --- | --- | --- | --- | --- | --- | --- | --- |
|  | ***N*** | ***%*** | ***N*** | % | ***N*** | % | ***N*** | % | ***N*** | % | ***N*** | % | ***N*** | % |
| **Gender** |  |  |  |  |  |  |  |  |  |  |  |  |  |  |
| Female | 2561 | 51.22 | 350 | 50.00 | 357 | 51.00 | 353 | 50.43 | 546 | 52.00 | 598 | 52.00 | 357 | 51.00 |
| Male | 2439 | 48.78 | 350 | 50.00 | 343 | 49.00 | 347 | 49.57 | 504 | 48.00 | 552 | 48.00 | 343 | 49.00 |
| Missing | 0 | 0.00 | 0 | 0.00 | 0 | 0.00 | 0 | 0.00 | 0 | 0.00 | 0.00 | 0 | 0 | 0.00 |
| Total | 5000 | 100.00 | 700 | 100.00 | 700 | 100.00 | 700 | 100.00 | 1050 | 100.00 | 100.00 | 100.00 | 700 | 100.00 |
|  |  |  |  |  |  |  |  |  |  |  |  |  |  |  |
| **Political ideology** |  |  |  |  |  |  |  |  |  |  |  |  |  |  |
| Very left wing/liberal | 286 | 5.72 | 29 | 4.14 | 57 | 8.14 | 53 | 7.57 | 35 | 3.33 | 43 | 3.74 | 69 | 9.86 |
| Left wing/liberal | 761 | 15.22 | 109 | 15.57 | 102 | 14.57 | 175 | 25.00 | 131 | 12.48 | 148 | 12.87 | 96 | 13.71 |
| Centre left/slightly liberal | 774 | 15.48 | 126 | 18.00 | 100 | 14.29 | 130 | 18.57 | 148 | 14.10 | 196 | 17.04 | 74 | 10.57 |
| Middle of the road | 1864 | 37.28 | 300 | 42.86 | 318 | 45.43 | 185 | 26.43 | 398 | 37.91 | 435 | 37.83 | 228 | 32.57 |
| Centre right/slightly conservative | 686 | 13.72 | 92 | 13.14 | 66 | 9.43 | 75 | 10.71 | 184 | 17.52 | 171 | 14.87 | 98 | 14.00 |
| Right wing/conservative | 463 | 9.26 | 32 | 4.57 | 40 | 5.71 | 62 | 8.86 | 126 | 12.00 | 126 | 10.96 | 77 | 11.00 |
| Very right wing/conservative | 143 | 2.86 | 10 | 1.43 | 11 | 1.57 | 16 | 2.29 | 23 | 2.19 | 28 | 2.44 | 55 | 7.86 |
| Missing | 23 | 0.46 | 2 | 0.29 | 6 | 0.86 | 4 | 0.57 | 5 | 0.48 | 3 | 0.26 | 3 | 0.43 |
| Total | 5000 | 100.00 | 700 | 100.00 | 700 | 100.00 | 700 | 100.00 | 1050 | 100.00 | 1150 | 100.00 | 700 | 100.00 |
|  |  |  |  |  |  |  |  |  |  |  |  |  |  |  |
| **Education** |  |  |  |  |  |  |  |  |  |  |  |  |  |  |
| No formal education above age 16 | 389 | 7.78 | 39 | 5.57 | 19 | 2.71 | 24 | 3.43 | 145 | 13.81 | 148 | 12.87 | 14 | 2.00 |
| Professional or technical qualifications above age 16 | 577 | 11.54 | 52 | 7.43 | 57 | 8.14 | 71 | 10.14 | 178 | 16.95 | 194 | 16.87 | 25 | 3.57 |
| School education up to age 18 | 1366 | 27.32 | 235 | 33.57 | 93 | 13.29 | 204 | 29.14 | 281 | 26.76 | 308 | 26.78 | 245 | 35.00 |
| Degree (Bachelors) or equivalent | 1965 | 39.30 | 265 | 37.86 | 457 | 65.29 | 285 | 40.71 | 314 | 29.91 | 342 | 29.74 | 302 | 43.14 |
| Degree (Masters) or other postgraduate qualification | 573 | 11.46 | 96 | 13.71 | 64 | 9.14 | 81 | 11.57 | 104 | 9.91 | 130 | 11.30 | 98 | 14.00 |
| Doctorate | 116 | 2.32 | 10 | 1.43 | 8 | 1.14 | 31 | 4.43 | 26 | 2.476 | 27 | 2.35 | 14 | 2.00 |
| Missing | 14 | 0.28 | 3 | 0.43 | 2 | 0.29 | 4 | 0.57 | 2 | 0.19 | 1 | 0.09 | 2 | 0.29 |
| Total | 5000 | 100.00 | 700 | 100.00 | 700 | 100.00 | 700 | 100.00 | 1050 | 100.00 | 1150 | 100.00 | 700 | 100.00 |
|  |  |  |  |  |  |  |  |  |  |  |  |  |  |  |
| **Minority status** |  |  |  |  |  |  |  |  |  |  |  |  |  |  |
| No | 3919 | 78.38 | 512 | 73.14 | 509 | 72.71 | 558 | 79.71 | 883 | 84.10 | 933 | 81.13 | 524 | 74.86 |
| Yes | 887 | 17.74 | 151 | 21.57 | 142 | 20.29 | 116 | 16.57 | 135 | 12.86 | 190 | 16.52 | 153 | 21.86 |
| Prefer not to answer | 178 | 3.56 | 36 | 5.14 | 47 | 6.71 | 21 | 3.00 | 30 | 2.86 | 24 | 2.09 | 20 | 2.86 |
| Missing | 16 | 0.32 | 1 | 0.14 | 2 | 0.29 | 5 | 0.71 | 2 | 0.19 | 3 | 0.26 | 3 | 0.43 |
| Total | 5000 | 100.00 | 700 | 100.00 | 700 | 100.00 | 700 | 100.00 | 1050 | 100.00 | 1150 | 100.00 | 700 | 100.00 |
|  |  |  |  |  |  |  |  |  |  |  |  |  |  |  |
| **Social media information exposure** |  |  |  |  |  |  |  |  |  |  |  |  |  |  |
| No | 1352 | 27.04 | 191 | 27.29 | 30 | 4.29 | 125 | 17.86 | 349 | 33.24 | 445 | 38.70 | 212 | 30.29 |
| Yes | 3642 | 72.84 | 508 | 72.57 | 670 | 95.71 | 575 | 82.14 | 700 | 66.67 | 702 | 61.04 | 487 | 69.57 |
| Missing | 6 | 0.12 | 1 | 0.14 | 0 | 0.00 | 0 | 0.00 | 1 | 0.10 | 3 | 0.26 | 1 | 0.14 |
| Total | 5000 | 100.00 | 700 | 100.00 | 700 | 100.00 | 700 | 100.00 | 1050 | 100.00 | 1150 | 100.00 | 700 | 100.00 |
|  |  |  |  |  |  |  |  |  |  |  |  |  |  |  |
| **WHO media exposure** |  |  |  |  |  |  |  |  |  |  |  |  |  |  |
| No | 947 | 18.94 | 70 | 10.00 | 40 | 5.71 | 76 | 10.86 | 272 | 25.91 | 320 | 27.83 | 169 | 24.14 |
| Yes | 4046 | 80.92 | 629 | 89.86 | 660 | 94.29 | 623 | 89.00 | 777 | 74.00 | 828 | 72.00 | 529 | 75.57 |
| Missing | 7 | 0.14 | 1 | 0.14 | 0 | 0.00 | 1 | 0.14 | 1 | 0.10 | 2 | 0.17 | 2 | 0.29 |
| Total | 5000 | 100.00 | 700 | 100.00 | 700 | 100.00 | 700 | 100.00 | 1050 | 100.00 | 1150 | 100.00 | 700 | 100.00 |
|  |  |  |  |  |  |  |  |  |  |  |  |  |  |  |
| **Vaccination - self^[[1]](#footnote-1)^** |  |  |  |  |  |  |  |  |  |  |  |  |  |  |
| No | 742 | 14.84 | - | - | 182 | 26.00 | 141 | 20.14 | - | - | 242 | 21.04 | 177 | 25.29 |
| Yes | 2501 | 50.02 | - | - | 516 | 73.71 | 558 | 79.71 | - | - | 905 | 78.70 | 522 | 74.57 |
| Missing | 1757 | 35.14 | - | - | 2 | 0.29 | 1 | 0.14 | - | - | 3 | 0.26 | 1 | 0.14 |
| Total | 5000 | 100.00 | - | - | 700 | 100.00 | 700 | 100.00 | - | - | 1150 | 100.00 | 700 | 100.00 |
|  |  |  |  |  |  |  |  |  |  |  |  |  |  |  |
| **Vaccination - recommend** |  |  |  |  |  |  |  |  |  |  |  |  |  |  |
| No | 612 | 12.24 | - | - | 170 | 24.29 | 122 | 17.43 | - | - | 181 | 15.74 | 139 | 19.86 |
| Yes | 2627 | 52.52 | - | - | 526 | 75.14 | 577 | 82.43 | - | - | 964 | 83.83 | 560 | 80.00 |
| Missing | 1761 | 35.22 | - | - | 4 | 0.57 | 1 | 0.14 | - | - | 5 | 0.44 | 1 | 0.14 |
| Total | 5000 | 100.00 | - | - | 700 | 100.00 | 700 | 100.00 | - | - | 1150 | 100.00 | 700 | 100.00 |

**Table S3**. Variables and items

| Type | Variable name | Example item | Scale |
| --- | --- | --- | --- |
| Psychological predictors | COVID-19 risk perception | How worried are you personally about the following issues at present? - Coronavirus/COVID-19 | 7 point Likert scale, 1 = not at all worried, 7 = very worried |
|  |  | How likely do you think it is that you will be directly and personally affected by the following in the next 6 months? - Catching the coronavirus/COVID-19 | 7 point Likert scale, 1= not at all likely, 7 = very likely |
|  |  | How likely do you think it is that your friends and family in the country you are currently living in will be directly affected by the following in the next 6 months? - Catching the coronavirus/COVID-19 | 7 point Likert scale, 1= not at all likely, 7 = very likely |
|  |  | How much do you agree or disagree with the following statements? - The coronavirus/COVID-19 will NOT affect very many people in the country I'm currently living in | Reverse coded, 5 point Likert scale, 1 = strongly disagree, 5 = strongly agree |
|  |  | How much do you agree or disagree with the following statements? - I will probably get sick with the coronavirus/COVID-19 | 5 point Likert scale, 1 = strongly disagree, 5 = strongly agree |
|  |  | How much do you agree or disagree with the following statements? - Getting sick with the coronavirus/COVID-19 can be serious | 5 point Likert scale, 1 = strongly disagree, 5 = strongly agree |
|  | Compliance with health guidance | Which of the following steps, if any, have you taken in the last month to prepare for the possibility of many cases of the coronavirus/COVID-19 in your community? Select all that apply. | 11 options, multiple options possible simultaneously: Washing your hands more often; Using alcohol-based hand sanitizer more often; Wearing a face mask; Avoiding social events (e.g., parties, family gatherings); Avoiding public transport; Eating out less at restaurants; Touching your face less; Shopping for groceries less often; Cooking at home more often; Staying home from work; Purchasing extra supplies or food |
|  | Trust in politicians’ approach effectiveness | How much do you trust the country’s politicians to deal effectively with the pandemic? | 7 point Likert scale, 1 = not at all, 7 = very much |
|  | Trust in WHO’s approach effectiveness | How much do you trust the World Health Organisation to know the best measures to take in the face of the pandemic? | 7 point Likert scale, 1 = not at all, 7 = very much |
|  | Trust in government | How much do you trust each of the following? - The current government of the country you are living in | 5 point Likert scale, 1 = Cannot be trusted at all to 5 = Can be trusted a lot |
|  | Trust in science | How much do you trust each of the following? – Scientists | 5 point Likert scale, 1 = cannot be trusted at all to 5 = can be trusted a lot |
|  | Trust in journalists | How much do you trust each of the following? - Journalists | 5 point Likert scale, 1 = cannot be trusted at all to 5 = can be trusted a lot |
|  | Getting information from social media | Have you come across information about coronavirus/COVID-19 from: social media or online blogs from individuals | Yes, no |
|  | Getting information from WHO | Have you come across information about coronavirus/COVID-19 from: the World Health Organization | Yes, no |
|  | Likelihood to vaccinate | If a vaccine were to be available for the coronavirus/COVID-19 now, would you – get vaccinated yourself? | Yes, no |
|  | Likelihood to recommend vaccination | If a vaccine were to be available for the coronavirus/COVID-19 now, would you - recommend vulnerable friends/family to get vaccinated? | Yes, no |
| Demographic  Predictors | Political ideology | Where do you feel your political views lie on a spectrum of left wing (or liberal) to right wing (or conservative)? | 7 point Likert scale, 1 = very left wing/ liberal, 7 = very right wing/ conservative |
|  | Gender | What is your gender? | Male, female, other, prefer not to say^2^ |
|  | Age | What is your age? | Free numeric text entry |
|  | Self-perceived minority status | Do you consider yourself to be part of a minority group within the country you are currently living in? | Yes, no, prefer not to answer |
|  | Education | Please indicate your highest educational qualification | No formal education above age 16  Professional or technical qualifications above age 16  School education up to age 18  Degree (Bachelors) or equivalent  Degree (Masters) or other postgraduate qualification  Doctorate |
|  | Numeracy | Out of 1,000 people in a small town 500 are members of a choir. Out of these 500 members in the choir 100 are men. Out of the 500 inhabitants that are not in the choir 300 are men. What is the probability that a randomly drawn man is a member of the choir? Please indicate the probability in percent. | Free numeric text entry |
|  |  | Imagine we are throwing a five-sided die 50 times. On average, out of these 50 throws how many times would this five-sided die show an odd number (1, 3 or 5)?____ out of 50 throws. | Free numeric text entry |
|  |  | Imagine we are throwing a loaded die (6 sides) 70 times. The probability that the die shows a 6 is twice as high as the probability of each of the other numbers. On average, out of these 70 throws how many times would the die show the number 6?____out of 70 throws. | Free numeric text entry |
|  |  | In a forest 20% of mushrooms are red, 50% brown and 30% white. A red mushroom is poisonous with a probability of 20%. A mushroom that is not red is poisonous with a probability of 5%. What is the probability that a poisonous mushroom in the forest is red?____ % | Free numeric text entry |
|  |  | Which represents the highest risk of something happening? 1 in 10 / 1 in 1000 / 1 in 100 | 3 multiple choice options |
|  |  | Imagine that we flip a fair coin 1,000 times. What is your best guess about how many times the coin would come up heads in 1,000 flips? | Free numeric text entry |
|  |  | In a scratch card lottery, the chance of winning a £10 prize on the card is 1%. What is your best guess about how many people would win a £10 prize if 1,000 people each buy a single scratch card? _____ person(s) out of 1,000. | Free numeric text entry |
|  |  | At a raffle, the chance of winning a car is 1 in 1,000. What percentage of tickets in the raffle win a car? ____%. | Free numeric text entry |

^2^ For analysis purposes, non-binary gender observations were removed

**Table S4.** Misinformation & factual items descriptive statistics

| **Country** | *Ireland* | | *Mexico* | | *Spain* | | *UK - April* | | *UK - May* | | *United States* | |
| --- | --- | --- | --- | --- | --- | --- | --- | --- | --- | --- | --- | --- |
|  | *M* | *SD* | *M* | *SD* | *M* | *SD* | *M* | *SD* | *M* | *SD* | *M* | *SD* |
| Misinformation | 2.49 | 1.34 | 2.79 | 1.38 | 2.67 | 1.34 | 2.31 | 1.25 | 2.32 | 1.30 | 2.36 | 1.28 |
| misinformation_bioengineering | 3.15 | 1.97 | 3.54 | 2.05 | 3.87 | 2.02 | 2.93 | 1.94 | 3.20 | 2.00 | 3.08 | 1.92 |
| misinformation_breath | 2.60 | 1.93 | 2.79 | 2.02 | 2.49 | 1.81 | 2.64 | 1.84 | 2.45 | 1.81 | 2.47 | 1.79 |
| misinformation_saltwater | 2.51 | 1.75 | 2.88 | 1.97 | 2.40 | 1.73 | 2.24 | 1.63 | 2.07 | 1.57 | 2.43 | 1.69 |
| misinformation_5g | 2.18 | 1.74 | 2.52 | 1.86 | 2.52 | 1.81 | 1.89 | 1.51 | 1.94 | 1.62 | 1.90 | 1.53 |
| misinformation_vaccination | 2.57 | 1.95 | 2.90 | 2.05 | 2.71 | 1.90 | 2.32 | 1.76 | 2.45 | 1.91 | 2.48 | 1.96 |
| misinformation_hot-air | 1.94 | 1.57 | 2.08 | 1.62 | 2.00 | 1.51 | 1.81 | 1.43 | 1.78 | 1.44 | 1.82 | 1.44 |
| factual_diabetes | 5.60 | 1.52 | 6.18 | 1.33 | 5.11 | 1.69 | 5.52 | 1.54 | 5.56 | 1.47 | 5.80 | 1.37 |
| factual_sanitizer | 5.20 | 1.72 | 4.80 | 1.99 | 5.01 | 1.72 | 4.89 | 1.73 | 4.99 | 1.69 | 5.02 | 1.62 |
| ambiguous_ibuprofen | 3.11 | 1.85 | 3.66 | 2.09 | 3.33 | 1.97 | 4.04 | 2.01 | 3.62 | 1.97 | 3.31 | 1.81 |

**Table S5.** Measures descriptive statistics

| **Variable** |  | *Overall* | *Ireland* | *Mexico* | *Spain* | *UK - April* | *UK - May* | *United States* |
| --- | --- | --- | --- | --- | --- | --- | --- | --- |
| Age | Valid | 4934 | 682 | 694 | 689 | 1039 | 1136 | 694 |
|  | Missing | 66 | 18 | 6 | 11 | 11 | 14 | 6 |
|  | Mean | 44.73 | 45.85 | 38.68 | 46.00 | 45.53 | 45.64 | 45.73 |
|  | Std. Dev. | 18.24 | 16.32 | 14.57 | 15.03 | 18.38 | 15.99 | 26.53 |
|  |  |  |  |  |  |  |  |  |
| Numeracy | Valid | 5000 | 700 | 700 | 700 | 1050 | 1150 | 700 |
|  | Missing | 0 | 0 | 0 | 0 | 0 | 0 | 0 |
|  | Mean | 2.69 | 2.58 | 2.41 | 2.46 | 2.85 | 2.83 | 2.86 |
|  | Std. Dev. | 1.35 | 1.35 | 1.27 | 1.28 | 1.35 | 1.39 | 1.33 |
|  |  |  |  |  |  |  |  |  |
| Compliance w/ health guidance | Valid | 5000 | 700 | 700 | 700 | 1050 | 1150 | 700 |
|  | Missing | 0 | 0 | 0 | 0 | 0 | 0 | 0 |
|  | Mean | 7.43 | 7.44 | 8.62 | 7.76 | 6.96 | 6.76 | 7.72 |
|  | Std. Dev. | 2.71 | 2.48 | 2.49 | 2.78 | 2.57 | 2.70 | 2.84 |
|  |  |  |  |  |  |  |  |  |
| COVID risk perception | Valid | 5000 | 700 | 700 | 700 | 1050 | 1150 | 700 |
|  | Missing | 0 | 0 | 0 | 0 | 0 | 0 | 0 |
|  | Mean | 4.34 | 4.34 | 4.36 | 4.42 | 4.46 | 4.26 | 4.19 |
|  | Std. Dev. | 0.84 | 0.79 | 0.92 | 0.79 | 0.77 | 0.81 | 0.94 |
|  |  |  |  |  |  |  |  |  |
| Trust in politicians' COVID approach | Valid | 4995 | 699 | 698 | 700 | 1050 | 1149 | 699 |
|  | Missing | 5 | 1 | 2 | 0 | 0 | 1 | 1 |
|  | Mean | 3.98 | 5.06 | 3.51 | 3.45 | 4.49 | 4.00 | 3.11 |
|  | Std. Dev. | 1.93 | 1.64 | 1.96 | 1.91 | 1.79 | 1.87 | 1.77 |
|  |  |  |  |  |  |  |  |  |
| Trust in WHO's COVID approach | Valid | 4993 | 699 | 698 | 700 | 1050 | 1148 | 698 |
|  | Missing | 7 | 1 | 2 | 0 | 0 | 2 | 2 |
|  | Mean | 5.02 | 5.42 | 5.58 | 4.88 | 5.04 | 4.76 | 4.57 |
|  | Std. Dev. | 1.68 | 1.49 | 1.62 | 1.62 | 1.56 | 1.69 | 1.90 |
|  |  |  |  |  |  |  |  |  |
| Trust in government | Valid | 4997 | 697 | 700 | 700 | 1050 | 1150 | 700 |
|  | Missing | 3 | 3 | 0 | 0 | 0 | 0 | 0 |
|  | Mean | 2.80 | 3.28 | 2.56 | 2.42 | 3.06 | 2.79 | 2.58 |
|  | Std. Dev. | 1.20 | 1.12 | 1.29 | 1.25 | 1.11 | 1.14 | 1.10 |
|  |  |  |  |  |  |  |  |  |
| Trust in scientists | Valid | 4993 | 697 | 700 | 698 | 1050 | 1148 | 700 |
|  | Missing | 7 | 3 | 0 | 2 | 0 | 2 | 0 |
|  | Mean | 3.91 | 3.91 | 4.11 | 4.06 | 3.85 | 3.76 | 3.87 |
|  | Std. Dev. | 0.91 | 0.86 | 0.96 | 0.92 | 0.87 | 0.92 | 0.90 |
|  |  |  |  |  |  |  |  |  |
| Trust in journalists | Valid | 4993 | 698 | 699 | 699 | 1050 | 1147 | 700 |
|  | Missing | 7 | 2 | 1 | 1 | 0 | 3 | 0 |
|  | Mean | 2.61 | 2.83 | 2.77 | 2.68 | 2.41 | 2.39 | 2.81 |
|  | Std. Dev. | 1.00 | 0.94 | 1.01 | 0.97 | 0.91 | 1.00 | 1.08 |

**Table S6.** Independent samples t-test – Misinformation & factual items, United Kingdom – April and May

|  | | | | | | | | | |
| --- | --- | --- | --- | --- | --- | --- | --- | --- | --- |
|  | | **t** | | **df** | | **p** | | **Cohen's d** | |
| Misinformation |  | -0.225 |  | 2198 |  | 0.822 |  | -0.010 |  |
| misinformation_bioengineering |  | -3.212 |  | 2198 |  | 0.001 |  | -0.137 |  |
| misinformation_vaccination |  | -1.667 |  | 2197 |  | 0.096 | ᵃ | -0.071 |  |
| misinformation_saltwater |  | 2.460 |  | 2196 |  | 0.014 | ᵃ | 0.105 |  |
| misinformation_5g |  | -0.862 |  | 2198 |  | 0.389 | ᵃ | -0.037 |  |
| misinformation_breath |  | 2.374 |  | 2196 |  | 0.018 |  | 0.101 |  |
| misinformation_hot-air |  | 0.606 |  | 2197 |  | 0.545 |  | 0.026 |  |
| factual_diabetes |  | -0.701 |  | 2197 |  | 0.483 |  | -0.030 |  |
| factual_sanitizer |  | -1.406 |  | 2198 |  | 0.160 |  | -0.060 |  |
| ambiguous_ibuprofen |  | 4.917 |  | 2198 |  | < .001 |  | 0.210 |  |
|  | | | | | | | | | |
| *Note:*  Student's t-test. | | | | | | | | | |
| ᵃ Levene's test is significant (*p* < .05), suggesting a violation of the equal variance assumption | | | | | | | | | |

**Table S7.** Tukey HSD pairwise comparisons by country

|  | | **Compared with** | | **Mean Difference** | | **SE** | | **t** | | **p_tukey_** | |
| --- | --- | --- | --- | --- | --- | --- | --- | --- | --- | --- | --- |
| Ireland |  | Mexico |  | -0.293 |  | 0.070 |  | -4.178 |  | < .001 |  |
|  |  | Spain |  | -0.172 |  | 0.070 |  | -2.449 |  | 0.103 |  |
|  |  | United Kingdom |  | 0.189 |  | 0.064 |  | 2.947 |  | 0.027 |  |
|  |  | United States |  | 0.131 |  | 0.070 |  | 1.869 |  | 0.334 |  |
| Mexico |  | Spain |  | 0.121 |  | 0.070 |  | 1.730 |  | 0.415 |  |
|  |  | United Kingdom |  | 0.482 |  | 0.064 |  | 7.526 |  | < .001 |  |
|  |  | United States |  | 0.424 |  | 0.070 |  | 6.047 |  | < .001 |  |
| Spain |  | United Kingdom |  | 0.360 |  | 0.064 |  | 5.633 |  | < .001 |  |
|  |  | United States |  | 0.303 |  | 0.070 |  | 4.319 |  | < .001 |  |
| United Kingdom |  | United States |  | -0.058 |  | 0.064 |  | -0.899 |  | 0.897 |  |
|  | | | | | | | | | | | |
| *Note:*  *p*-value adjusted for comparing a family of 5. | | | | | | | | | | | |

| **Table S8.** Misinformation items frequency tables | | | | | | | | | | | | | |
| --- | --- | --- | --- | --- | --- | --- | --- | --- | --- | --- | --- | --- | --- |
| **M_Bioengineering** | *Ireland* | | *Mexico* | | *Spain* | | *UK - April* | | *UK - May* | | *USA* | | |
|  | *N* | *%* | *N* | *%* | *N* | *%* | *N* | *%* | *N* | *%* | | *N* | *%* |
| 1 | 226 | 32.29 | 182 | 26.00 | 139 | 19.86 | 389 | 37.05 | 366 | 31.83 | | 227 | 32.43 |
| 2 | 91 | 13.00 | 79 | 11.29 | 67 | 9.57 | 153 | 14.57 | 151 | 13.13 | | 94 | 13.43 |
| 3 | 64 | 9.14 | 67 | 9.57 | 70 | 10.00 | 86 | 8.19 | 103 | 8.96 | | 73 | 10.43 |
| 4 | 136 | 19.43 | 141 | 20.14 | 161 | 23.00 | 181 | 17.24 | 215 | 18.70 | | 150 | 21.43 |
| 5 | 82 | 11.71 | 90 | 12.86 | 92 | 13.14 | 113 | 10.76 | 137 | 11.91 | | 62 | 8.86 |
| 6 | 47 | 6.71 | 58 | 8.29 | 74 | 10.57 | 60 | 5.71 | 86 | 7.48 | | 44 | 6.29 |
| 7 | 52 | 7.43 | 82 | 11.71 | 97 | 13.86 | 68 | 6.48 | 92 | 8.00 | | 49 | 7.00 |
| Missing | 2 | 0.29 | 1 | 0.14 | 0 | 0.00 | 0 | 0.00 | 0 | 0.00 | | 1 | 0.14 |
| Total | 700 | 100.00 | 700 | 100.00 | 700 | 100.00 | 1050 | 100.00 | 1150 | 100.00 | | 700 | 100.00 |
|  |  |  |  |  |  |  |  |  |  |  | |  |  |
| **M_Vaccination** | *Ireland* | | *Mexico* | | *Spain* | | *UK - April* | | *UK - May* | | | *USA* | |
|  | *N* | *%* | *N* | *%* | *N* | *%* | *N* | *%* | *N* | *%* | | *N* | *%* |
| 1 | 349 | 49.86 | 290 | 41.4 | 308 | 44.00 | 561 | 53.43 | 605 | 52.61 | | 375 | 53.57 |
| 2 | 74 | 10.57 | 82 | 11.7 | 72 | 10.29 | 123 | 11.71 | 130 | 11.30 | | 70 | 10.00 |
| 3 | 49 | 7.00 | 55 | 7.9 | 78 | 11.14 | 88 | 8.38 | 77 | 6.70 | | 47 | 6.71 |
| 4 | 99 | 14.14 | 114 | 16.3 | 110 | 15.71 | 141 | 13.43 | 152 | 13.22 | | 80 | 11.43 |
| 5 | 46 | 6.57 | 52 | 7.4 | 57 | 8.14 | 53 | 5.05 | 67 | 5.83 | | 55 | 7.86 |
| 6 | 38 | 5.43 | 43 | 6.1 | 38 | 5.43 | 45 | 4.29 | 51 | 4.44 | | 23 | 3.29 |
| 7 | 42 | 6.00 | 61 | 8.7 | 37 | 5.29 | 38 | 3.62 | 68 | 5.91 | | 49 | 7.00 |
| Missing | 3 | 0.43 | 3 | 0.4 | 0 | 0.00 | 1 | 0.10 | 0 | 0.00 | | 1 | 0.14 |
| Total | 700 | 100.00 | 700 | 100.0 | 700 | 100.00 | 1050 | 100.00 | 1150 | 100.00 | | 700 | 100.00 |
|  |  |  |  |  |  |  |  |  |  |  | |  |  |
| **M_Salt water** | *Ireland* | | *Mexico* | | *Spain* | | *UK - April* | | *UK - May* | | | *USA* | |
|  | *N* | *%* | *N* | *%* | *N* | *%* | *N* | *%* | *N* | *%* | | *N* | *%* |
| 1 | 326 | 46.57 | 283 | 40.43 | 354 | 50.57 | 546 | 52.00 | 663 | 57.65 | | 309 | 44.14 |
| 2 | 90 | 12.86 | 81 | 11.57 | 76 | 10.86 | 156 | 14.86 | 161 | 14.00 | | 136 | 19.43 |
| 3 | 56 | 8.00 | 66 | 9.43 | 65 | 9.29 | 92 | 8.76 | 93 | 8.09 | | 58 | 8.29 |
| 4 | 125 | 17.86 | 112 | 16.00 | 103 | 14.71 | 141 | 13.43 | 110 | 9.57 | | 100 | 14.29 |
| 5 | 56 | 8.00 | 66 | 9.43 | 60 | 8.57 | 60 | 5.71 | 70 | 6.09 | | 50 | 7.14 |
| 6 | 25 | 3.57 | 48 | 6.86 | 24 | 3.43 | 30 | 2.86 | 31 | 2.70 | | 26 | 3.71 |
| 7 | 21 | 3.00 | 43 | 6.14 | 18 | 2.57 | 25 | 2.38 | 20 | 1.74 | | 20 | 2.86 |
| Missing | 1 | 0.14 | 1 | 0.14 | 0 | 0.00 | 0 | 0.00 | 2 | 0.17 | | 1 | 0.14 |
| Total | 700 | 100.00 | 700 | 100.00 | 700 | 100.00 | 1050 | 100.00 | 1150 | 100.00 | | 700 | 100.00 |
|  |  |  |  |  |  |  |  |  |  |  | |  |  |
| **M_5G** | *Ireland* | | *Mexico* | | *Spain* | | *UK - April* | | *UK - May* | | | *USA* | |
|  | *N* | *%* | *N* | *%* | *N* | *%* | *N* | *%* | *N* | *%* | | *N* | *%* |
| 1 | 420 | 60.00 | 347 | 49.57 | 339 | 48.43 | 703 | 66.95 | 774 | 67.30 | | 460 | 65.71 |
| 2 | 63 | 9.00 | 77 | 11.00 | 74 | 10.57 | 96 | 9.14 | 104 | 9.04 | | 77 | 11.00 |
| 3 | 35 | 5.00 | 48 | 6.86 | 75 | 10.71 | 68 | 6.48 | 47 | 4.09 | | 32 | 4.57 |
| 4 | 100 | 14.29 | 112 | 16.00 | 93 | 13.29 | 97 | 9.24 | 107 | 9.30 | | 74 | 10.57 |
| 5 | 36 | 5.14 | 54 | 7.71 | 58 | 8.29 | 43 | 4.10 | 53 | 4.61 | | 27 | 3.86 |
| 6 | 19 | 2.71 | 27 | 3.86 | 36 | 5.14 | 23 | 2.19 | 36 | 3.13 | | 14 | 2.00 |
| 7 | 26 | 3.71 | 34 | 4.86 | 24 | 3.43 | 20 | 1.91 | 29 | 2.52 | | 15 | 2.14 |
| Missing | 1 | 0.14 | 1 | 0.14 | 1 | 0.14 | 0 | 0.00 | 0 | 0.00 | | 1 | 0.14 |
| Total | 700 | 100.00 | 700 | 100.00 | 700 | 100.00 | 1050 | 100.00 | 1150 | 100.00 | | 700 | 100.00 |
|  |  |  |  |  |  |  |  |  |  |  | |  |  |
| **M_Breath** | *Ireland* | | *Mexico* | | *Spain* | | *UK - April* | | *UK - May* | | | *USA* | |
|  | *N* | *%* | *N* | *%* | *N* | *%* | *N* | *%* | *N* | *%* | | *N* | *%* |
| 1 | 338 | 48.29 | 306 | 43.71 | 346 | 49.43 | 459 | 43.71 | 574 | 49.91 | | 338 | 48.29 |
| 2 | 74 | 10.57 | 90 | 12.86 | 78 | 11.14 | 137 | 13.05 | 145 | 12.61 | | 94 | 13.43 |
| 3 | 58 | 8.29 | 52 | 7.43 | 59 | 8.43 | 111 | 10.57 | 89 | 7.74 | | 58 | 8.29 |
| 4 | 102 | 14.57 | 92 | 13.14 | 105 | 15.00 | 151 | 14.38 | 165 | 14.35 | | 96 | 13.71 |
| 5 | 47 | 6.71 | 62 | 8.86 | 58 | 8.29 | 85 | 8.10 | 77 | 6.70 | | 61 | 8.71 |
| 6 | 38 | 5.43 | 46 | 6.57 | 27 | 3.86 | 65 | 6.19 | 55 | 4.78 | | 27 | 3.86 |
| 7 | 40 | 5.71 | 51 | 7.29 | 27 | 3.86 | 40 | 3.81 | 45 | 3.91 | | 25 | 3.57 |
| Missing | 3 | 0.43 | 1 | 0.14 | 0 | 0.00 | 2 | 0.19 | 0 | 0.00 | | 1 | 0.14 |
| Total | 700 | 100.00 | 700 | 100.00 | 700 | 100.00 | 1050 | 100.00 | 1150 | 100.00 | | 700 | 100.00 |
|  |  |  |  |  |  |  |  |  |  |  | |  |  |
| **M_Hot air** | *Ireland* | | *Mexico* | | *Spain* | | *UK - April* | | *UK - May* | | | *USA* | |
|  | *N* | *%* | *N* | *%* | *N* | *%* | *N* | *%* | *N* | *%* | | *N* | *%* |
| 1 | 457 | 65.29 | 414 | 59.14 | 429 | 61.29 | 699 | 66.57 | 805 | 70.00 | | 466 | 66.57 |
| 2 | 72 | 10.29 | 89 | 12.71 | 75 | 10.71 | 131 | 12.48 | 115 | 10.00 | | 90 | 12.86 |
| 3 | 34 | 4.86 | 48 | 6.86 | 55 | 7.86 | 65 | 6.19 | 52 | 4.52 | | 40 | 5.71 |
| 4 | 68 | 9.71 | 75 | 10.71 | 79 | 11.29 | 81 | 7.71 | 92 | 8.00 | | 51 | 7.29 |
| 5 | 34 | 4.86 | 33 | 4.71 | 38 | 5.43 | 40 | 3.81 | 45 | 3.91 | | 24 | 3.43 |
| 6 | 19 | 2.71 | 21 | 3.00 | 17 | 2.43 | 13 | 1.24 | 22 | 1.91 | | 17 | 2.43 |
| 7 | 14 | 2.00 | 18 | 2.57 | 7 | 1.00 | 21 | 2.00 | 18 | 1.57 | | 11 | 1.57 |
| Missing | 2 | 0.29 | 2 | 0.29 | 0 | 0.00 | 0 | 0.00 | 1 | 0.09 | | 1 | 0.14 |
| Total | 700 | 100.00 | 700 | 100.00 | 700 | 100.00 | 1050 | 100.00 | 1150 | 100.00 | | 700 | 100.00 |
|  |  |  |  |  |  |  |  |  |  |  | |  |  |
| **F_Diabetes** | *Ireland* | | *Mexico* | | *Spain* | | *UK - April* | | *UK - May* | | | *USA* | |
|  | *N* | *%* | *N* | *%* | *N* | *%* | *N* | *%* | *N* | *%* | | *N* | *%* |
| 1 | 14 | 2.00 | 13 | 1.86 | 33 | 4.71 | 29 | 2.76 | 27 | 2.35 | | 8 | 1.14 |
| 2 | 17 | 2.43 | 9 | 1.29 | 18 | 2.57 | 30 | 2.86 | 18 | 1.57 | | 13 | 1.86 |
| 3 | 34 | 4.86 | 13 | 1.86 | 65 | 9.29 | 50 | 4.76 | 45 | 3.91 | | 21 | 3.00 |
| 4 | 102 | 14.57 | 49 | 7.00 | 131 | 18.71 | 124 | 11.81 | 172 | 14.96 | | 79 | 11.29 |
| 5 | 113 | 16.14 | 51 | 7.29 | 133 | 19.00 | 206 | 19.62 | 230 | 20.00 | | 123 | 17.57 |
| 6 | 141 | 20.14 | 148 | 21.14 | 117 | 16.71 | 247 | 23.52 | 245 | 21.30 | | 161 | 23.00 |
| 7 | 276 | 39.43 | 416 | 59.43 | 203 | 29.00 | 363 | 34.57 | 413 | 35.91 | | 294 | 42.00 |
| Missing | 3 | 0.43 | 1 | 0.14 | 0 | 0.00 | 1 | 0.10 | 0 | 0.00 | | 1 | 0.14 |
| Total | 700 | 100.00 | 700 | 100.00 | 700 | 100.00 | 1050 | 100.00 | 1150 | 100.00 | | 700 | 100.00 |
|  |  |  |  |  |  |  |  |  |  |  | |  |  |
| **F_Sanitizer** | *Ireland* | | *Mexico* | | *Spain* | | *UK - April* | | *UK - May* | | | *USA* | |
|  | *N* | *%* | *N* | *%* | *N* | *%* | *N* | *%* | *N* | *%* | | *N* | *%* |
| 1 | 38 | 5.43 | 78 | 11.14 | 37 | 5.29 | 59 | 5.62 | 61 | 5.30 | | 25 | 3.57 |
| 2 | 28 | 4.00 | 42 | 6.00 | 28 | 4.00 | 54 | 5.14 | 47 | 4.09 | | 34 | 4.86 |
| 3 | 36 | 5.14 | 50 | 7.14 | 60 | 8.57 | 101 | 9.62 | 96 | 8.35 | | 59 | 8.43 |
| 4 | 107 | 15.29 | 100 | 14.29 | 132 | 18.86 | 184 | 17.52 | 192 | 16.70 | | 120 | 17.14 |
| 5 | 146 | 20.86 | 103 | 14.71 | 129 | 18.43 | 209 | 19.91 | 255 | 22.17 | | 163 | 23.29 |
| 6 | 128 | 18.29 | 146 | 20.86 | 138 | 19.71 | 215 | 20.48 | 235 | 20.44 | | 141 | 20.14 |
| 7 | 215 | 30.71 | 178 | 25.43 | 176 | 25.14 | 228 | 21.71 | 264 | 22.96 | | 157 | 22.43 |
| Missing | 2 | 0.29 | 3 | 0.43 | 0 | 0.00 | 0 | 0.00 | 0 | 0.00 | | 1 | 0.14 |
| Total | 700 | 100.00 | 700 | 100.00 | 700 | 100.00 | 1050 | 100.00 | 1150 | 100.00 | | 700 | 100.00 |
|  |  |  |  |  |  |  |  |  |  |  | |  |  |
| **A_Ibuprofen** | *Ireland* | | *Mexico* | | *Spain* | | *UK - April* | | *UK - May* | | | *USA* | |
|  | *N* | *%* | *N* | *%* | *N* | *%* | *N* | *%* | *N* | *%* | | *N* | *%* |
| 1 | 204 | 29.14 | 164 | 23.43 | 198 | 28.29 | 188 | 17.91 | 256 | 22.26 | | 166 | 23.71 |
| 2 | 97 | 13.86 | 86 | 12.29 | 75 | 10.71 | 86 | 8.19 | 131 | 11.39 | | 103 | 14.71 |
| 3 | 87 | 12.43 | 74 | 10.57 | 97 | 13.86 | 119 | 11.33 | 141 | 12.26 | | 81 | 11.57 |
| 4 | 158 | 22.57 | 142 | 20.29 | 139 | 19.86 | 200 | 19.05 | 232 | 20.17 | | 173 | 24.71 |
| 5 | 65 | 9.29 | 75 | 10.71 | 68 | 9.71 | 181 | 17.24 | 164 | 14.26 | | 91 | 13.00 |
| 6 | 45 | 6.43 | 51 | 7.29 | 65 | 9.29 | 112 | 10.67 | 106 | 9.22 | | 45 | 6.43 |
| 7 | 40 | 5.71 | 107 | 15.29 | 58 | 8.29 | 164 | 15.62 | 120 | 10.44 | | 40 | 5.71 |
| Missing | 4 | 0.57 | 1 | 0.14 | 0 | 0.00 | 0 | 0.00 | 0 | 0.00 | | 1 | 0.14 |
| Total | 700 | 100.00 | 700 | 100.00 | 700 | 100.00 | 1050 | 100.00 | 1150 | 100.00 | | 700 | 100.00 |

| **Model Summary – Would you get vaccinated against COVID-19 yourself** | | | | | | | | | | | | | | | | | | | | | |
| --- | --- | --- | --- | --- | --- | --- | --- | --- | --- | --- | --- | --- | --- | --- | --- | --- | --- | --- | --- | --- | --- |
| *Model* | | *Deviance* | | *AIC* | | *BIC* | | *df* | | *Χ²* | | *p* | | *McFadden R²* | | *Nagelkerke R²* | | *Tjur R²* | | *Cox & Snell R²* | |
| H₀ |  | 3302.253 |  | 3304.253 |  | 3310.289 |  | 3090 |  |  |  |  |  |  |  |  |  |  |  |  |  |
| H₁ |  | 3023.539 |  | 3039.539 |  | 3087.829 |  | 3083 |  | 278.714 |  | < .001 |  | 0.084 |  | 0.086 |  | 0.027 |  | 0.086 |  |
|  | | | | | | | | | | | | | | | | | | | | | |

**Table S9.** Logit regression model results for vaccine hesitancy (2x)

| **Model Summary – Would you recommend vulnerable friends or family members to get vaccinated against COVID-19** | | | | | | | | | | | | | | | | | | | | | |
| --- | --- | --- | --- | --- | --- | --- | --- | --- | --- | --- | --- | --- | --- | --- | --- | --- | --- | --- | --- | --- | --- |
| *Model* | | *Deviance* | | *AIC* | | *BIC* | | *df* | | *Χ²* | | *p* | | *McFadden R²* | | *Nagelkerke R²* | | *Tjur R²* | | *Cox & Snell R²* | |
| H₀ |  | 2960.175 |  | 2962.175 |  | 2968.211 |  | 3088 |  |  |  |  |  |  |  |  |  |  |  |  |  |
| H₁ |  | 2674.351 |  | 2690.351 |  | 2738.636 |  | 3081 |  | 285.825 |  | < .001 |  | 0.097 |  | 0.088 |  | 0.003 |  | 0.088 |  |
|  | | | | | | | | | | | | | | | | | | | | | |

**Table S10.** Logistic regression model for vaccine hesitancy, by country.

|  |  | **Would you get vaccinated against COVID yourself** | | | | | | **Would you recommend others to get vaccinated** | | | | | |
| --- | --- | --- | --- | --- | --- | --- | --- | --- | --- | --- | --- | --- | --- |
| **Country** | **Variable** | *Estimate* | *Std. Error* | *z* | *p* | *OR* | 95%CI | *Estimate* | *Std. Error* | *z* | *p* | *OR* | 95%CI |
| *Pooled* | (Intercept) | -0.38 | 0.34 | -1.12 | 0.262 | 0.69 | 0.354-1.325 | 0.03 | 0.36 | 0.09 | 0.93 | 1.03 | 0.512-2.081 |
|  | Age | 0.01 | 0.00 | 3.37 | 0.001 | **1.01** | 1.004-1.016 | 0.01 | 0.00 | 2.25 | 0.024 | **1.01** | 1.001-1.014 |
|  | Gender | -0.38 | 0.09 | -4.12 | <0.001 | **0.68** | 0.57-0.819 | -0.31 | 0.10 | -3.09 | 0.002 | **0.74** | 0.605-0.893 |
|  | Education | 0.03 | 0.04 | 0.75 | 0.451 | 1.03 | 0.95-1.121 | 0.00 | 0.05 | 0.08 | 0.936 | 1.00 | 0.918-1.097 |
|  | Political ideology | -0.04 | 0.03 | -1.25 | 0.21 | 0.96 | 0.903-1.023 | -0.01 | 0.03 | -0.34 | 0.737 | 0.99 | 0.924-1.057 |
|  | Self-perceived minority status | 0.05 | 0.12 | 0.39 | 0.697 | 1.05 | 0.835-1.317 | 0.06 | 0.13 | 0.47 | 0.64 | 1.06 | 0.833-1.357 |
|  | Numeracy | 0.00 | 0.04 | -0.08 | 0.94 | 1.00 | 0.928-1.072 | 0.00 | 0.04 | 0.06 | 0.956 | 1.00 | 0.927-1.083 |
|  | Trust in scientists | 0.55 | 0.05 | 11.31 | <0.001 | **1.73** | 1.577-1.908 | 0.58 | 0.05 | 11.25 | <0.001 | **1.79** | 1.614-1.975 |
|  | Misinformation | -0.26 | 0.04 | -7.20 | <0.001 | **0.77** | 0.717-0.827 | -0.33 | 0.04 | -8.46 | <0.001 | **0.72** | 0.669-0.778 |
|  |  |  |  |  |  |  |  |  |  |  |  |  |  |
| *Mexico* | (Intercept) | -0.32 | 0.71 | -0.45 | 0.655 | 0.73 | 0.183-2.93 | -0.10 | 0.72 | -0.14 | 0.89 | 0.91 | 0.221-3.743 |
|  | Age | -0.01 | 0.01 | -0.75 | 0.454 | 1.00 | 0.982-1.008 | -0.01 | 0.01 | -1.07 | 0.284 | 0.99 | 0.98-1.006 |
|  | Gender | -0.72 | 0.20 | -3.68 | <0.001 | **0.49** | 0.331-0.712 | -0.55 | 0.20 | -2.77 | 0.006 | **0.58** | 0.391-0.85 |
|  | Education | 0.06 | 0.11 | 0.52 | 0.602 | 1.06 | 0.853-1.308 | 0.06 | 0.11 | 0.54 | 0.587 | 1.06 | 0.853-1.316 |
|  | Political ideology | 0.17 | 0.07 | 2.33 | 0.02 | **1.18** | 1.028-1.367 | 0.11 | 0.07 | 1.54 | 0.125 | 1.12 | 0.969-1.296 |
|  | Self-perceived minority status | -0.01 | 0.23 | -0.04 | 0.971 | 0.99 | 0.636-1.571 | -0.06 | 0.23 | -0.25 | 0.806 | 0.95 | 0.603-1.503 |
|  | Numeracy | -0.06 | 0.08 | -0.69 | 0.49 | 0.95 | 0.808-1.106 | -0.03 | 0.08 | -0.37 | 0.713 | 0.97 | 0.826-1.138 |
|  | Trust in scientists | 0.49 | 0.10 | 4.91 | <0.001 | **1.64** | 1.347-1.999 | 0.51 | 0.10 | 4.99 | <0.001 | **1.66** | 1.362-2.028 |
|  | Misinformation | -0.24 | 0.07 | -3.19 | 0.001 | **0.79** | 0.683-0.913 | -0.26 | 0.08 | -3.42 | 0.001 | **0.78** | 0.669-0.897 |
|  |  |  |  |  |  |  |  |  |  |  |  |  |  |
| *Spain* | (Intercept) | -0.91 | 0.77 | -1.18 | 0.239 | 0.40 | 0.088-1.82 | 0.32 | 0.81 | 0.40 | 0.69 | 1.38 | 0.282-6.82 |
|  | Age | 0.00 | 0.01 | 0.63 | 0.529 | 1.00 | 0.991-1.018 | 0.00 | 0.01 | 0.02 | 0.981 | 1.00 | 0.986-1.015 |
|  | Gender | -0.49 | 0.21 | -2.38 | 0.017 | **0.61** | 0.406-0.914 | -0.41 | 0.22 | -1.87 | 0.061 | 0.66 | 0.429-1.017 |
|  | Education | 0.09 | 0.10 | 0.90 | 0.371 | 1.09 | 0.903-1.313 | -0.09 | 0.10 | -0.89 | 0.372 | 0.91 | 0.745-1.114 |
|  | Political ideology | -0.03 | 0.07 | -0.40 | 0.691 | 0.97 | 0.852-1.114 | -0.06 | 0.07 | -0.84 | 0.4 | 0.94 | 0.817-1.085 |
|  | Self-perceived minority status | 0.18 | 0.28 | 0.62 | 0.532 | 1.19 | 0.699-2.098 | 0.10 | 0.29 | 0.34 | 0.735 | 1.10 | 0.633-2.001 |
|  | Numeracy | 0.02 | 0.09 | 0.20 | 0.839 | 1.02 | 0.858-1.207 | 0.04 | 0.09 | 0.44 | 0.658 | 1.04 | 0.869-1.248 |
|  | Trust in scientists | 0.60 | 0.11 | 5.54 | <0.001 | **1.83** | 1.479-2.268 | 0.59 | 0.11 | 5.22 | <0.001 | **1.81** | 1.45-2.267 |
|  | Misinformation | -0.11 | 0.08 | -1.29 | 0.197 | 0.90 | 0.767-1.058 | -0.14 | 0.09 | -1.65 | 0.098 | 0.87 | 0.733-1.028 |
|  |  |  |  |  |  |  |  |  |  |  |  |  |  |
| *UK - May* | (Intercept) | -0.55 | 0.59 | -0.94 | 0.346 | 0.58 | 0.182-1.813 | -0.61 | 0.65 | -0.94 | 0.348 | 0.55 | 0.153-1.93 |
|  | Age | 0.02 | 0.01 | 3.56 | <0.001 | **1.02** | 1.009-1.031 | 0.01 | 0.01 | 2.13 | 0.034 | **1.01** | 1.001-1.026 |
|  | Gender | -0.13 | 0.16 | -0.80 | 0.425 | 0.88 | 0.641-1.204 | -0.03 | 0.18 | -0.19 | 0.853 | 0.97 | 0.676-1.38 |
|  | Education | 0.03 | 0.07 | 0.47 | 0.638 | 1.03 | 0.907-1.173 | 0.04 | 0.07 | 0.50 | 0.617 | 1.04 | 0.898-1.199 |
|  | Political ideology | -0.01 | 0.06 | -0.20 | 0.84 | 0.99 | 0.878-1.112 | 0.09 | 0.07 | 1.38 | 0.169 | 1.10 | 0.961-1.257 |
|  | Self-perceived minority status | 0.05 | 0.21 | 0.25 | 0.803 | 1.05 | 0.707-1.591 | -0.02 | 0.23 | -0.07 | 0.944 | 0.98 | 0.636-1.552 |
|  | Numeracy | 0.03 | 0.06 | 0.43 | 0.666 | 1.03 | 0.909-1.159 | -0.02 | 0.07 | -0.28 | 0.778 | 0.98 | 0.855-1.123 |
|  | Trust in scientists | 0.47 | 0.08 | 5.56 | <0.001 | **1.60** | 1.354-1.883 | 0.63 | 0.09 | 6.71 | <0.001 | **1.87** | 1.56-2.252 |
|  | Misinformation | -0.30 | 0.07 | -4.66 | <0.001 | **0.74** | 0.65-0.839 | -0.37 | 0.07 | -5.04 | <0.001 | **0.69** | 0.602-0.8 |
|  |  |  |  |  |  |  |  |  |  |  |  |  |  |
| *USA* | (Intercept) | -1.03 | 0.79 | -1.30 | 0.195 | 0.36 | 0.074-1.678 | -0.82 | 0.85 | -0.96 | 0.336 | 0.44 | 0.081-2.331 |
|  | Age | 0.01 | 0.01 | 1.79 | 0.073 | 1.01 | 0.999-1.025 | 0.01 | 0.01 | 1.64 | 0.101 | 1.01 | 0.998-1.026 |
|  | Gender | -0.38 | 0.20 | -1.89 | 0.059 | 0.69 | 0.465-1.013 | -0.43 | 0.22 | -1.95 | 0.051 | 0.65 | 0.423-1 |
|  | Education | 0.18 | 0.11 | 1.55 | 0.122 | 1.19 | 0.954-1.493 | 0.32 | 0.13 | 2.59 | 0.01 | 1.38 | 1.083-1.768 |
|  | Political ideology | -0.23 | 0.07 | -3.46 | 0.001 | **0.79** | 0.693-0.903 | -0.19 | 0.07 | -2.52 | 0.012 | **0.83** | 0.718-0.959 |
|  | Self-perceived minority status | -0.13 | 0.25 | -0.55 | 0.584 | 0.88 | 0.543-1.424 | 0.09 | 0.28 | 0.32 | 0.752 | 1.09 | 0.642-1.897 |
|  | Numeracy | 0.03 | 0.08 | 0.32 | 0.75 | 1.03 | 0.875-1.202 | -0.01 | 0.09 | -0.08 | 0.934 | 0.99 | 0.834-1.179 |
|  | Trust in scientists | 0.72 | 0.12 | 6.08 | <0.001 | **2.05** | 1.632-2.595 | 0.70 | 0.13 | 5.59 | <0.001 | **2.02** | 1.584-2.599 |
|  | Misinformation | -0.23 | 0.08 | -2.72 | 0.007 | **0.80** | 0.678-0.939 | -0.39 | 0.09 | -4.32 | <0.001 | **0.68** | 0.568-0.808 |

*Note:* OR stands for Odds Ratios. Standard errors are calculated using profiled log-likelihood.

**Table S11.** Variance Inflation Factors per variable, pooled and by country.

| **Variable** | *Pooled* | *Ireland* | *Mexico* | *Spain* | *UK* | *USA* |
| --- | --- | --- | --- | --- | --- | --- |
| Age | 1.09 | 1.28 | 1.02 | 1.1 | 1.15 | 1.05 |
| Female | 1.03 | 1.13 | 1.03 | 1.04 | 1.06 | 1.03 |
| Education | 1.09 | 1.13 | 1.04 | 1.04 | 1.10 | 1.11 |
| Politically right wing | 1.12 | 1.09 | 1.06 | 1.29 | 1.38 | 1.59 |
| Self-perceived minority status | 1.05 | 1.11 | 1.03 | 1.06 | 1.10 | 1.07 |
| Numeracy | 1.07 | 1.12 | 1.07 | 1.06 | 1.11 | 1.13 |
| Trust in scientists | 1.37 | 1.30 | 1.37 | 1.25 | 1.39 | 1.66 |
| Trust in government | 1.92 | 1.70 | 1.82 | 2.07 | 2.08 | 1.77 |
| Trust in journalists | 1.22 | 1.23 | 1.16 | 1.25 | 1.17 | 1.59 |
| COVID risk perception | 1.12 | 1.10 | 1.11 | 1.12 | 1.14 | 1.34 |
| Trust in politicians' COVID approach | 1.93 | 1.79 | 1.71 | 1.89 | 2.06 | 1.75 |
| Trust in WHO's COVID approach | 1.51 | 1.56 | 1.45 | 1.37 | 1.47 | 1.82 |
| Getting information from social media | 1.15 | 1.11 | 1.05 | 1.21 | 1.20 | 1.10 |
| Getting information from WHO | 1.19 | 1.10 | 1.08 | 1.19 | 1.20 | 1.20 |

|  | *Pooled* | *Ireland* | *Mexico* | *Spain* | *UK (April)* | *United States* |
| --- | --- | --- | --- | --- | --- | --- |
| (Intercept) | 4.11 *** | 4.80 *** | 3.53 *** | 4.19 *** | 4.24 *** | 3.52 *** |
|  | [3.77, 4.45] | [3.91, 5.70] | [2.64, 4.41] | [3.30, 5.07] | [3.48, 5.00] | [2.80, 4.25] |
| Age | **-0.01 ***** | **-0.02 ***** | **0.01 *** | **-0.01 **** | **-0.01 **** | **-0.00 *** |
|  | [-0.02, -0.01] | [-0.03, -0.01] | [0.00, 0.01] | [-0.02, -0.00] | [-0.03, -0.00] | [-0.01, -0.00] |
| Female | **-0.16 ***** | -0.02 | -0.05 | **-0.28 **** | -0.01 | -0.19 * |
|  | [-0.23, -0.10] | [-0.21, 0.17] | [-0.24, 0.15] | [-0.47, -0.09] | [-0.15, 0.13] | [-0.34, -0.03] |
| Education | 0.03 * | 0.05 | 0.03 | 0.03 | -0.02 | 0.03 |
|  | [0.00, 0.06] | [-0.04, 0.14] | [-0.09, 0.14] | [-0.06, 0.12] | [-0.08, 0.05] | [-0.07, 0.12] |
| `Politically right wing` | **0.06 ***** | **0.15 ***** | 0.09 * | **0.13 ***** | 0.03 | 0.03 |
|  | [0.03, 0.09] | [0.08, 0.22] | [0.02, 0.17] | [0.06, 0.20] | [-0.03, 0.10] | [-0.03, 0.09] |
| `Self-perceived minority status` | **0.38 ***** | **0.47 ***** | **0.41 **** | **0.34 *** | 0.12 | **0.34 **** |
|  | [0.28, 0.48] | [0.23, 0.71] | [0.15, 0.67] | [0.07, 0.61] | [-0.11, 0.34] | [0.14, 0.55] |
| Numeracy | **-0.30 ***** | **-0.23 ***** | **-0.25 ***** | **-0.30 ***** | **-0.28 ***** | **-0.26 ***** |
|  | [-0.32, -0.27] | [-0.31, -0.16] | [-0.33, -0.17] | [-0.38, -0.22] | [-0.34, -0.23] | [-0.33, -0.19] |
| `Trust in scientists` | **-0.26 ***** | **-0.33 ***** | **-0.17 **** | **-0.25 ***** | **-0.34 ***** | **-0.33 ***** |
|  | [-0.31, -0.21] | [-0.46, -0.20] | [-0.30, -0.05] | [-0.37, -0.12] | [-0.44, -0.23] | [-0.46, -0.21] |
| `Trust in government` | -0.01 | -0.03 | 0.00 | -0.04 | 0.00 | 0.11 * |
|  | [-0.04, 0.03] | [-0.13, 0.08] | [-0.09, 0.10] | [-0.15, 0.07] | [-0.09, 0.09] | [0.02, 0.20] |
| `Trust in journalists` | **0.11 ***** | 0.04 | **0.11 *** | **0.13 *** | **0.13 **** | 0.02 |
|  | [0.07, 0.15] | [-0.08, 0.16] | [0.01, 0.22] | [0.02, 0.24] | [0.04, 0.22] | [-0.08, 0.12] |
| `COVID risk perception` | -0.03 | -0.07 | -0.04 | -0.07 | 0.04 | -0.03 |
|  | [-0.07, 0.02] | [-0.20, 0.06] | [-0.16, 0.08] | [-0.20, 0.07] | [-0.06, 0.14] | [-0.14, 0.07] |
| `Trust in politicians' COVID approach` | **0.07 ***** | -0.01 | **0.15 ***** | **0.08 *** | 0.05 | **0.19 ***** |
|  | [0.05, 0.10] | [-0.08, 0.06] | [0.09, 0.22] | [0.00, 0.15] | [-0.01, 0.10] | [0.13, 0.26] |
| `Trust in WHO's COVID approach` | **-0.05 ***** | -0.00 | **-0.18 ***** | 0.03 | -0.02 | -0.02 |
|  | [-0.07, -0.02] | [-0.07, 0.07] | [-0.25, -0.10] | [-0.03, 0.10] | [-0.07, 0.03] | [-0.08, 0.04] |
| `Getting information from social media` | 0.35 *** | 0.25 ** | 0.27 | 0.19 | 0.31 *** | 0.34 *** |
|  | [0.27, 0.43] | [0.06, 0.45] | [-0.23, 0.76] | [-0.06, 0.44] | [0.14, 0.48] | [0.16, 0.51] |
| `Getting information from WHO` | **-0.14 **** | -0.29 | -0.20 | **-0.42 **** | -0.13 | -0.11 |
|  | [-0.23, -0.04] | [-0.59, 0.01] | [-0.64, 0.24] | [-0.73, -0.11] | [-0.30, 0.04] | [-0.33, 0.12] |
| *** *p* < 0.001; ** *p* < 0.01; * *p* < 0.05. | | | | | | |

**Table S12.** Robustness check with heteroscedastic robust standard errors. Significant predictors are marked in bold.

.

**Table S13.** Correlation matrix – pooled sample. Means, standard deviations, and correlations with confidence intervals.

|  | *M* | *SD* | 1 | 2 | 3 | 4 | 5 | 6 | 7 | 8 | 9 | 10 | 11 | |
| --- | --- | --- | --- | --- | --- | --- | --- | --- | --- | --- | --- | --- | --- | --- |
|  |  |  |  |  |  |  |  |  |  |  |  |  |  | |
| 1. Misinformation | 2.46 | 1.32 |  |  |  |  |  |  |  |  |  |  |  | |
|  |  |  |  |  |  |  |  |  |  |  |  |  |  | |
| 2. Age | 44.73 | 18.24 | -.19** |  |  |  |  |  |  |  |  |  |  | |
|  |  |  | [-.22, -.16] |  |  |  |  |  |  |  |  |  |  | |
|  |  |  |  |  |  |  |  |  |  |  |  |  |  | |
| 3. Numeracy | 2.69 | 1.35 | -.35** | .01 |  |  |  |  |  |  |  |  |  | |
|  |  |  | [-.37, -.32] | [-.02, .03] |  |  |  |  |  |  |  |  |  | |
|  |  |  |  |  |  |  |  |  |  |  |  |  |  | |
| 4. Political ideology | 3.78 | 1.42 | .07** | .13** | -.00 |  |  |  |  |  |  |  |  | |
|  |  |  | [.04, .10] | [.10, .15] | [-.03, .02] |  |  |  |  |  |  |  |  | |
|  |  |  |  |  |  |  |  |  |  |  |  |  |  | |
| 5. Education | 3.42 | 1.15 | .01 | -.13** | .13** | -.07** |  |  |  |  |  |  |  | |
|  |  |  | [-.02, .03] | [-.16, -.11] | [.10, .15] | [-.10, -.04] |  |  |  |  |  |  |  | |
|  |  |  |  |  |  |  |  |  |  |  |  |  |  | |
| 6. Compliance | 7.43 | 2.71 | -.12** | .07** | .09** | -.01 | .11** |  |  |  |  |  |  | |
|  |  |  | [-.15, -.10] | [.04, .09] | [.06, .12] | [-.04, .02] | [.08, .13] |  |  |  |  |  |  | |
|  |  |  |  |  |  |  |  |  |  |  |  |  |  | |
| 7. Trust in pols' COVID approach | 3.98 | 1.93 | .07** | .06** | -.04** | .14** | -.08** | -.00 |  |  |  |  |  | |
|  |  |  | [.04, .09] | [.03, .09] | [-.07, -.01] | [.11, .16] | [-.11, -.05] | [-.03, .02] |  |  |  |  |  | |
|  |  |  |  |  |  |  |  |  |  |  |  |  |  | |
| 8. Trust in WHO COVID approach | 5.02 | 1.68 | -.12** | .00 | .03* | -.17** | .03* | .21** | .30** |  |  |  |  | |
|  |  |  | [-.15, -.09] | [-.03, .03] | [.00, .06] | [-.20, -.15] | [.01, .06] | [.18, .24] | [.27, .32] |  |  |  |  | |
|  |  |  |  |  |  |  |  |  |  |  |  |  |  | |
| 9. Trust in scientists | 3.91 | 0.91 | -.22** | .02 | .13** | -.11** | .11** | .19** | .13** | .42** |  |  |  | |
|  |  |  | [-.24, -.19] | [-.00, .05] | [.10, .16] | [-.14, -.08] | [.08, .14] | [.17, .22] | [.11, .16] | [.40, .45] |  |  |  | |
|  |  |  |  |  |  |  |  |  |  |  |  |  |  | |
| 10. Trust in journalists | 2.61 | 1.00 | .04** | .02 | -.05** | -.11** | .12** | .06** | .12** | .28** | .28** |  |  | |
|  |  |  | [.02, .07] | [-.00, .05] | [-.08, -.02] | [-.14, -.09] | [.09, .14] | [.03, .09] | [.09, .15] | [.26, .31] | [.26, .31] |  |  | |
|  |  |  |  |  |  |  |  |  |  |  |  |  |  | |
| 11. Trust in government | 2.80 | 1.20 | .04** | .05** | -.02 | .15** | -.05** | -.01 | .65** | .19** | .20** | .23** |  | |
|  |  |  | [.02, .07] | [.03, .08] | [-.05, .00] | [.12, .18] | [-.08, -.02] | [-.04, .02] | [.63, .66] | [.16, .22] | [.17, .23] | [.21, .26] |  | |
|  |  |  |  |  |  |  |  |  |  |  |  |  |  | |
| 12. COVID risk perception | 4.34 | 0.84 | -.09** | .04** | .07** | -.04** | .03* | .33** | .05** | .26** | .22** | .08** | -.01 | |
|  |  |  | [-.12, -.06] | [.01, .07] | [.05, .10] | [-.07, -.01] | [.00, .06] | [.30, .35] | [.02, .08] | [.23, .28] | [.20, .25] | [.05, .10] | [-.04, .02] |  |
|  |  |  |  |  |  |  |  |  |  |  |  |  |  |  |

*Note.* *M* and *SD* are used to represent mean and standard deviation, respectively. Values in square brackets indicate the 95% confidence interval for each correlation. The confidence interval is a plausible range of population correlations that could have caused the sample correlation (Cumming, 2014). * indicates *p* < .05, ** indicates *p* < .01.

**Table S14.** Correlation matrix – Ireland. Means, standard deviations, and correlations with confidence intervals.

| Variable | *M* | *SD* | 1 | 2 | 3 | 4 | 5 | 6 | 7 | 8 | 9 | 10 | 11 |
| --- | --- | --- | --- | --- | --- | --- | --- | --- | --- | --- | --- | --- | --- |
|  |  |  |  |  |  |  |  |  |  |  |  |  |  |
| 1. Misinformation | 2.49 | 1.34 |  |  |  |  |  |  |  |  |  |  |  |
|  |  |  |  |  |  |  |  |  |  |  |  |  |  |
| 2. Age | 45.85 | 16.32 | -.31** |  |  |  |  |  |  |  |  |  |  |
|  |  |  | [-.38, -.24] |  |  |  |  |  |  |  |  |  |  |
|  |  |  |  |  |  |  |  |  |  |  |  |  |  |
| 3. Numeracy | 2.58 | 1.35 | -.29** | -.04 |  |  |  |  |  |  |  |  |  |
|  |  |  | [-.36, -.22] | [-.12, .03] |  |  |  |  |  |  |  |  |  |
|  |  |  |  |  |  |  |  |  |  |  |  |  |  |
| 4. Political ideology | 3.65 | 1.23 | .13** | .12** | -.05 |  |  |  |  |  |  |  |  |
|  |  |  | [.06, .20] | [.04, .19] | [-.12, .03] |  |  |  |  |  |  |  |  |
|  |  |  |  |  |  |  |  |  |  |  |  |  |  |
| 5. Education | 3.51 | 1.05 | .07 | -.25** | .18** | -.03 |  |  |  |  |  |  |  |
|  |  |  | [-.00, .14] | [-.32, -.18] | [.10, .25] | [-.10, .05] |  |  |  |  |  |  |  |
|  |  |  |  |  |  |  |  |  |  |  |  |  |  |
| 6. Compliance | 7.44 | 2.48 | -.13** | .12** | .14** | -.03 | -.02 |  |  |  |  |  |  |
|  |  |  | [-.21, -.06] | [.05, .20] | [.06, .21] | [-.10, .05] | [-.09, .06] |  |  |  |  |  |  |
|  |  |  |  |  |  |  |  |  |  |  |  |  |  |
| 7. Trust in pols' COVID approach | 5.06 | 1.64 | -.15** | .19** | .06 | .11** | -.02 | .14** |  |  |  |  |  |
|  |  |  | [-.23, -.08] | [.12, .27] | [-.02, .13] | [.04, .19] | [-.10, .05] | [.07, .21] |  |  |  |  |  |
|  |  |  |  |  |  |  |  |  |  |  |  |  |  |
| 8. Trust in WHO COVID approach | 5.42 | 1.49 | -.21** | .22** | .05 | -.07 | -.07 | .14** | .50** |  |  |  |  |
|  |  |  | [-.28, -.14] | [.14, .29] | [-.03, .12] | [-.14, .00] | [-.14, .01] | [.07, .21] | [.45, .56] |  |  |  |  |
|  |  |  |  |  |  |  |  |  |  |  |  |  |  |
| 9. Trust in scientists | 3.91 | 0.86 | -.28** | .10* | .11** | -.01 | .01 | .11** | .34** | .37** |  |  |  |
|  |  |  | [-.35, -.21] | [.02, .17] | [.04, .18] | [-.09, .06] | [-.07, .08] | [.04, .18] | [.27, .40] | [.30, .43] |  |  |  |
|  |  |  |  |  |  |  |  |  |  |  |  |  |  |
| 10. Trust in journalists | 2.83 | 0.94 | -.04 | .07 | -.01 | .02 | .05 | -.02 | .26** | .23** | .27** |  |  |
|  |  |  | [-.12, .03] | [-.01, .14] | [-.09, .06] | [-.05, .09] | [-.02, .12] | [-.09, .06] | [.19, .33] | [.15, .29] | [.20, .34] |  |  |
|  |  |  |  |  |  |  |  |  |  |  |  |  |  |
| 11. Trust in government | 3.28 | 1.12 | -.10** | .15** | .00 | .16** | .02 | .06 | .55** | .33** | .34** | .37** |  |
|  |  |  | [-.17, -.03] | [.07, .22] | [-.07, .08] | [.09, .23] | [-.06, .09] | [-.01, .14] | [.49, .60] | [.26, .39] | [.27, .40] | [.31, .44] |  |
|  |  |  |  |  |  |  |  |  |  |  |  |  |  |
| 12. COVID risk perception | 4.34 | 0.79 | -.11** | .03 | .15** | -.00 | .07 | .31** | .07 | .17** | .16** | .01 | .03 |
|  |  |  | [-.19, -.04] | [-.05, .10] | [.07, .22] | [-.08, .07] | [-.00, .14] | [.24, .37] | [-.01, .14] | [.10, .24] | [.08, .23] | [-.06, .09] | [-.04, .10] |

*Note.* *M* and *SD* are used to represent mean and standard deviation, respectively. Values in square brackets indicate the 95% confidence interval for each correlation. The confidence interval is a plausible range of population correlations that could have caused the sample correlation (Cumming, 2014). * indicates *p* < .05, ** indicates *p* < .01.

**Table S15.** Correlation matrix – Mexico. Means, standard deviations, and correlations with confidence intervals.

| Variable | *M* | *SD* | 1 | 2 | 3 | 4 | 5 | 6 | 7 | 8 | 9 | 10 | 11 |  |
| --- | --- | --- | --- | --- | --- | --- | --- | --- | --- | --- | --- | --- | --- | --- |
|  |  |  |  |  |  |  |  |  |  |  |  |  |  |  |
| 1. Misinformation | 2.79 | 1.38 |  |  |  |  |  |  |  |  |  |  |  | |
|  |  |  |  |  |  |  |  |  |  |  |  |  |  | |
| 2. Age | 38.68 | 14.56 | .11** |  |  |  |  |  |  |  |  |  |  | |
|  |  |  | [.03, .18] |  |  |  |  |  |  |  |  |  |  | |
|  |  |  |  |  |  |  |  |  |  |  |  |  |  | |
| 3. Numeracy | 2.41 | 1.27 | -.29** | -.03 |  |  |  |  |  |  |  |  |  | |
|  |  |  | [-.36, -.22] | [-.11, .04] |  |  |  |  |  |  |  |  |  | |
|  |  |  |  |  |  |  |  |  |  |  |  |  |  | |
| 4. Political ideology | 3.57 | 1.33 | .05 | .09* | -.00 |  |  |  |  |  |  |  |  | |
|  |  |  | [-.03, .12] | [.02, .16] | [-.08, .07] |  |  |  |  |  |  |  |  | |
|  |  |  |  |  |  |  |  |  |  |  |  |  |  | |
| 5. Education | 3.74 | 0.88 | -.04 | .03 | .12** | -.01 |  |  |  |  |  |  |  | |
|  |  |  | [-.11, .04] | [-.04, .11] | [.04, .19] | [-.08, .07] |  |  |  |  |  |  |  | |
|  |  |  |  |  |  |  |  |  |  |  |  |  |  | |
| 6. Compliance | 8.62 | 2.49 | -.18** | .17** | .09* | .05 | .04 |  |  |  |  |  |  | |
|  |  |  | [-.25, -.11] | [.10, .24] | [.02, .17] | [-.02, .12] | [-.03, .12] |  |  |  |  |  |  | |
|  |  |  |  |  |  |  |  |  |  |  |  |  |  | |
| 7. Trust in pols' COVID approach | 3.51 | 1.96 | .13** | -.01 | .04 | -.10** | -.02 | .02 |  |  |  |  |  | |
|  |  |  | [.05, .20] | [-.09, .06] | [-.04, .11] | [-.17, -.03] | [-.09, .05] | [-.05, .10] |  |  |  |  |  | |
|  |  |  |  |  |  |  |  |  |  |  |  |  |  | |
| 8. Trust in WHO COVID approach | 5.58 | 1.62 | -.25** | -.02 | .13** | .05 | .03 | .30** | .24** |  |  |  |  | |
|  |  |  | [-.31, -.17] | [-.09, .05] | [.06, .21] | [-.03, .12] | [-.05, .10] | [.24, .37] | [.17, .31] |  |  |  |  | |
|  |  |  |  |  |  |  |  |  |  |  |  |  |  | |
| 9. Trust in scientists | 4.11 | 0.96 | -.21** | .01 | .15** | .02 | .12** | .16** | .15** | .43** |  |  |  | |
|  |  |  | [-.28, -.14] | [-.07, .08] | [.08, .22] | [-.06, .09] | [.05, .20] | [.09, .23] | [.07, .22] | [.37, .49] |  |  |  | |
|  |  |  |  |  |  |  |  |  |  |  |  |  |  | |
| 10. Trust in journalists | 2.77 | 1.01 | .04 | .06 | .03 | .07 | .05 | .04 | .13** | .24** | .26** |  |  | |
|  |  |  | [-.04, .11] | [-.01, .14] | [-.05, .10] | [-.01, .14] | [-.03, .12] | [-.03, .12] | [.05, .20] | [.17, .31] | [.19, .33] |  |  | |
|  |  |  |  |  |  |  |  |  |  |  |  |  |  | |
| 11. Trust in government | 2.56 | 1.29 | .06 | .02 | .01 | -.14** | -.01 | .02 | .61** | .20** | .25** | .23** |  | |
|  |  |  | [-.01, .13] | [-.05, .10] | [-.06, .09] | [-.22, -.07] | [-.09, .06] | [-.05, .09] | [.56, .65] | [.13, .27] | [.18, .32] | [.16, .30] |  | |
|  |  |  |  |  |  |  |  |  |  |  |  |  |  | |
| 12. COVID risk perception | 4.35 | 0.92 | -.13** | -.01 | .11** | .08* | .06 | .32** | .01 | .29** | .17** | .12** | -.01 | |
|  |  |  | [-.20, -.06] | [-.08, .06] | [.04, .19] | [.00, .15] | [-.01, .14] | [.25, .39] | [-.07, .08] | [.23, .36] | [.10, .24] | [.04, .19] | [-.08, .07] | |

*Note.* *M* and *SD* are used to represent mean and standard deviation, respectively. Values in square brackets indicate the 95% confidence interval for each correlation. The confidence interval is a plausible range of population correlations that could have caused the sample correlation (Cumming, 2014). * indicates *p* < .05, ** indicates *p* < .01.

**Table S16.** Correlation matrix – Spain. Means, standard deviations, and correlations with confidence intervals.

| Variable | *M* | *SD* | 1 | 2 | 3 | 4 | 5 | 6 | 7 | 8 | 9 | 10 | 11 |
| --- | --- | --- | --- | --- | --- | --- | --- | --- | --- | --- | --- | --- | --- |
|  |  |  |  |  |  |  |  |  |  |  |  |  |  |
| 1. Misinformation | 2.67 | 1.34 |  |  |  |  |  |  |  |  |  |  |  |
|  |  |  |  |  |  |  |  |  |  |  |  |  |  |
| 2. Age | 46.00 | 15.03 | -.14** |  |  |  |  |  |  |  |  |  |  |
|  |  |  | [-.21, -.07] |  |  |  |  |  |  |  |  |  |  |
|  |  |  |  |  |  |  |  |  |  |  |  |  |  |
| 3. Numeracy | 2.46 | 1.28 | -.32** | .06 |  |  |  |  |  |  |  |  |  |
|  |  |  | [-.39, -.26] | [-.02, .13] |  |  |  |  |  |  |  |  |  |
|  |  |  |  |  |  |  |  |  |  |  |  |  |  |
| 4. Political ideology | 3.44 | 1.49 | .12** | .10* | -.03 |  |  |  |  |  |  |  |  |
|  |  |  | [.05, .20] | [.02, .17] | [-.11, .04] |  |  |  |  |  |  |  |  |
|  |  |  |  |  |  |  |  |  |  |  |  |  |  |
| 5. Education | 3.60 | 1.07 | .02 | -.05 | .03 | .08* |  |  |  |  |  |  |  |
|  |  |  | [-.05, .10] | [-.13, .02] | [-.05, .10] | [.01, .16] |  |  |  |  |  |  |  |
|  |  |  |  |  |  |  |  |  |  |  |  |  |  |
| 6. Compliance | 7.76 | 2.78 | -.13** | .05 | .06 | .12** | .04 |  |  |  |  |  |  |
|  |  |  | [-.21, -.06] | [-.02, .13] | [-.01, .14] | [.05, .20] | [-.04, .11] |  |  |  |  |  |  |
|  |  |  |  |  |  |  |  |  |  |  |  |  |  |
| 7. Trust in pols' COVID approach | 3.45 | 1.91 | .06 | -.04 | .00 | -.34** | -.03 | -.05 |  |  |  |  |  |
|  |  |  | [-.02, .13] | [-.11, .04] | [-.07, .08] | [-.40, -.27] | [-.11, .04] | [-.12, .02] |  |  |  |  |  |
|  |  |  |  |  |  |  |  |  |  |  |  |  |  |
| 8. Trust in WHO COVID approach | 4.88 | 1.62 | -.05 | -.00 | .06 | -.15** | -.02 | .16** | .36** |  |  |  |  |
|  |  |  | [-.12, .03] | [-.08, .07] | [-.01, .14] | [-.22, -.08] | [-.09, .06] | [.09, .23] | [.29, .42] |  |  |  |  |
|  |  |  |  |  |  |  |  |  |  |  |  |  |  |
| 9. Trust in scientists | 4.06 | 0.92 | -.19** | .05 | .11** | -.07 | .07 | .14** | .09* | .35** |  |  |  |
|  |  |  | [-.26, -.11] | [-.02, .13] | [.04, .18] | [-.15, .00] | [-.01, .14] | [.06, .21] | [.01, .16] | [.29, .42] |  |  |  |
|  |  |  |  |  |  |  |  |  |  |  |  |  |  |
| 10. Trust in journalists | 2.68 | 0.96 | .10** | .05 | -.06 | -.02 | .03 | -.01 | .24** | .20** | .21** |  |  |
|  |  |  | [.03, .17] | [-.03, .12] | [-.13, .02] | [-.10, .05] | [-.04, .11] | [-.08, .07] | [.17, .31] | [.13, .27] | [.13, .28] |  |  |
|  |  |  |  |  |  |  |  |  |  |  |  |  |  |
| 11. Trust in government | 2.42 | 1.25 | .04 | -.07 | -.04 | -.39** | -.00 | -.07 | .63** | .28** | .13** | .38** |  |
|  |  |  | [-.04, .11] | [-.14, .01] | [-.12, .03] | [-.45, -.33] | [-.08, .07] | [-.15, .00] | [.58, .67] | [.21, .35] | [.06, .20] | [.31, .44] |  |
|  |  |  |  |  |  |  |  |  |  |  |  |  |  |
| 12. COVID risk perception | 4.42 | 0.79 | -.09* | .06 | .08* | .13** | .03 | .28** | -.06 | .16** | .19** | -.03 | -.13** |
|  |  |  | [-.16, -.01] | [-.01, .14] | [.00, .15] | [.06, .21] | [-.04, .11] | [.21, .35] | [-.13, .01] | [.09, .23] | [.11, .26] | [-.10, .05] | [-.20, -.06] |

*Note.* *M* and *SD* are used to represent mean and standard deviation, respectively. Values in square brackets indicate the 95% confidence interval for each correlation. The confidence interval is a plausible range of population correlations that could have caused the sample correlation (Cumming, 2014). * indicates *p* < .05, ** indicates *p* < .01.

**Table S17.** Correlation matrix – United Kingdom (April). Means, standard deviations, and correlations with confidence intervals.

| Variable | *M* | *SD* | 1 | 2 | 3 | 4 | 5 | 6 | 7 | 8 | 9 | 10 | 11 |
| --- | --- | --- | --- | --- | --- | --- | --- | --- | --- | --- | --- | --- | --- |
|  |  |  |  |  |  |  |  |  |  |  |  |  |  |
| 1. Misinformation | 2.31 | 1.25 |  |  |  |  |  |  |  |  |  |  |  |
|  |  |  |  |  |  |  |  |  |  |  |  |  |  |
| 2. Age | 45.53 | 18.38 | -.24** |  |  |  |  |  |  |  |  |  |  |
|  |  |  | [-.29, -.18] |  |  |  |  |  |  |  |  |  |  |
|  |  |  |  |  |  |  |  |  |  |  |  |  |  |
| 3. Numeracy | 2.85 | 1.35 | -.35** | .00 |  |  |  |  |  |  |  |  |  |
|  |  |  | [-.40, -.29] | [-.06, .06] |  |  |  |  |  |  |  |  |  |
|  |  |  |  |  |  |  |  |  |  |  |  |  |  |
| 4. Political ideology | 3.99 | 1.34 | .02 | .15** | .00 |  |  |  |  |  |  |  |  |
|  |  |  | [-.04, .08] | [.09, .20] | [-.06, .06] |  |  |  |  |  |  |  |  |
|  |  |  |  |  |  |  |  |  |  |  |  |  |  |
| 5. Education | 3.13 | 1.28 | -.04 | -.18** | .16** | -.11** |  |  |  |  |  |  |  |
|  |  |  | [-.10, .02] | [-.24, -.12] | [.10, .21] | [-.17, -.05] |  |  |  |  |  |  |  |
|  |  |  |  |  |  |  |  |  |  |  |  |  |  |
| 6. Compliance | 6.96 | 2.57 | -.05 | .08* | .11** | .06* | .08* |  |  |  |  |  |  |
|  |  |  | [-.11, .01] | [.02, .14] | [.05, .17] | [.00, .12] | [.02, .14] |  |  |  |  |  |  |
|  |  |  |  |  |  |  |  |  |  |  |  |  |  |
| 7. Trust in pols' COVID approach | 4.49 | 1.79 | .01 | .15** | -.02 | .36** | -.13** | .08** |  |  |  |  |  |
|  |  |  | [-.05, .07] | [.09, .21] | [-.08, .04] | [.30, .41] | [-.18, -.07] | [.02, .14] |  |  |  |  |  |
|  |  |  |  |  |  |  |  |  |  |  |  |  |  |
| 8. Trust in WHO COVID approach | 5.04 | 1.56 | -.06 | .00 | .04 | -.11** | -.02 | .15** | .36** |  |  |  |  |
|  |  |  | [-.12, .00] | [-.06, .06] | [-.02, .10] | [-.17, -.05] | [-.08, .04] | [.09, .21] | [.31, .41] |  |  |  |  |
|  |  |  |  |  |  |  |  |  |  |  |  |  |  |
| 9. Trust in scientists | 3.85 | 0.87 | -.27** | .02 | .21** | -.03 | .07* | .11** | .22** | .36** |  |  |  |
|  |  |  | [-.32, -.21] | [-.04, .08] | [.15, .27] | [-.09, .03] | [.01, .13] | [.05, .17] | [.16, .27] | [.31, .42] |  |  |  |
|  |  |  |  |  |  |  |  |  |  |  |  |  |  |
| 10. Trust in journalists | 2.41 | 0.91 | .05 | .05 | -.05 | -.10** | .06 | -.01 | .13** | .20** | .18** |  |  |
|  |  |  | [-.01, .11] | [-.01, .11] | [-.11, .01] | [-.16, -.04] | [-.01, .12] | [-.07, .05] | [.07, .19] | [.14, .26] | [.12, .24] |  |  |
|  |  |  |  |  |  |  |  |  |  |  |  |  |  |
| 11. Trust in government | 3.06 | 1.11 | -.02 | .12** | -.01 | .39** | -.09** | .08* | .63** | .20** | .30** | .25** |  |
|  |  |  | [-.08, .04] | [.06, .18] | [-.07, .05] | [.34, .44] | [-.15, -.03] | [.02, .14] | [.59, .67] | [.14, .25] | [.25, .36] | [.19, .30] |  |
|  |  |  |  |  |  |  |  |  |  |  |  |  |  |
| 12. COVID risk perception | 4.46 | 0.77 | -.04 | .06 | .10** | -.02 | .01 | .36** | .09** | .21** | .21** | .02 | .04 |
|  |  |  | [-.10, .02] | [-.00, .12] | [.04, .16] | [-.09, .04] | [-.05, .07] | [.30, .41] | [.03, .15] | [.15, .27] | [.15, .27] | [-.04, .08] | [-.02, .10] |

*Note.* *M* and *SD* are used to represent mean and standard deviation, respectively. Values in square brackets indicate the 95% confidence interval for each correlation. The confidence interval is a plausible range of population correlations that could have caused the sample correlation (Cumming, 2014). * indicates *p* < .05, ** indicates *p* < .01.

**Table S18.** Correlation matrix – United Kingdom (May). Means, standard deviations, and correlations with confidence intervals.

| Variable | *M* | *SD* | 1 | 2 | 3 | 4 | 5 | 6 | 7 | 8 | 9 | 10 | 11 |
| --- | --- | --- | --- | --- | --- | --- | --- | --- | --- | --- | --- | --- | --- |
|  |  |  |  |  |  |  |  |  |  |  |  |  |  |
| 1. Misinformation | 2.32 | 1.30 |  |  |  |  |  |  |  |  |  |  |  |
|  |  |  |  |  |  |  |  |  |  |  |  |  |  |
| 2. Age | 45.64 | 15.99 | -.28** |  |  |  |  |  |  |  |  |  |  |
|  |  |  | [-.34, -.23] |  |  |  |  |  |  |  |  |  |  |
|  |  |  |  |  |  |  |  |  |  |  |  |  |  |
| 3. Numeracy | 2.83 | 1.39 | -.37** | -.01 |  |  |  |  |  |  |  |  |  |
|  |  |  | [-.42, -.32] | [-.07, .05] |  |  |  |  |  |  |  |  |  |
|  |  |  |  |  |  |  |  |  |  |  |  |  |  |
| 4. Political ideology | 3.90 | 1.35 | .03 | .21** | -.04 |  |  |  |  |  |  |  |  |
|  |  |  | [-.02, .09] | [.15, .26] | [-.10, .01] |  |  |  |  |  |  |  |  |
|  |  |  |  |  |  |  |  |  |  |  |  |  |  |
| 5. Education | 3.17 | 1.27 | -.02 | -.23** | .19** | -.06* |  |  |  |  |  |  |  |
|  |  |  | [-.08, .04] | [-.28, -.17] | [.14, .25] | [-.12, -.00] |  |  |  |  |  |  |  |
|  |  |  |  |  |  |  |  |  |  |  |  |  |  |
| 6. Compliance | 6.76 | 2.70 | -.23** | .08** | .17** | .02 | .07* |  |  |  |  |  |  |
|  |  |  | [-.28, -.17] | [.02, .14] | [.11, .22] | [-.04, .08] | [.01, .13] |  |  |  |  |  |  |
|  |  |  |  |  |  |  |  |  |  |  |  |  |  |
| 7. Trust in pols' COVID approach | 3.99 | 1.86 | .08** | .10** | -.11** | .34** | -.01 | .05 |  |  |  |  |  |
|  |  |  | [.02, .14] | [.05, .16] | [-.16, -.05] | [.28, .39] | [-.07, .05] | [-.01, .11] |  |  |  |  |  |
|  |  |  |  |  |  |  |  |  |  |  |  |  |  |
| 8. Trust in WHO COVID approach | 4.76 | 1.69 | -.15** | .03 | .06* | -.17** | .10** | .18** | .29** |  |  |  |  |
|  |  |  | [-.21, -.09] | [-.03, .08] | [.01, .12] | [-.22, -.11] | [.04, .16] | [.12, .23] | [.24, .34] |  |  |  |  |
|  |  |  |  |  |  |  |  |  |  |  |  |  |  |
| 9. Trust in scientists | 3.76 | 0.92 | -.20** | .00 | .18** | -.09** | .10** | .23** | .21** | .47** |  |  |  |
|  |  |  | [-.26, -.15] | [-.06, .06] | [.12, .23] | [-.15, -.03] | [.04, .16] | [.17, .28] | [.15, .26] | [.42, .51] |  |  |  |
|  |  |  |  |  |  |  |  |  |  |  |  |  |  |
| 10. Trust in journalists | 2.39 | 1.00 | .09** | -.08* | -.05 | -.10** | .14** | -.02 | .15** | .28** | .27** |  |  |
|  |  |  | [.03, .15] | [-.13, -.02] | [-.11, .01] | [-.16, -.04] | [.08, .19] | [-.08, .03] | [.09, .20] | [.23, .33] | [.21, .32] |  |  |
|  |  |  |  |  |  |  |  |  |  |  |  |  |  |
| 11. Trust in government | 2.79 | 1.14 | .10** | .09** | -.06* | .36** | .02 | .06* | .68** | .18** | .26** | .28** |  |
|  |  |  | [.04, .15] | [.04, .15] | [-.12, -.00] | [.31, .41] | [-.04, .08] | [.00, .12] | [.65, .71] | [.12, .23] | [.20, .31] | [.22, .33] |  |
|  |  |  |  |  |  |  |  |  |  |  |  |  |  |
| 12. COVID risk perception | 4.26 | 0.81 | -.08** | .06* | .06* | -.01 | .01 | .35** | .12** | .23** | .23** | .01 | .04 |
|  |  |  | [-.14, -.02] | [.00, .12] | [.00, .12] | [-.07, .04] | [-.05, .06] | [.30, .40] | [.06, .18] | [.18, .29] | [.17, .28] | [-.05, .06] | [-.02, .10] |

*Note.* *M* and *SD* are used to represent mean and standard deviation, respectively. Values in square brackets indicate the 95% confidence interval for each correlation. The confidence interval is a plausible range of population correlations that could have caused the sample correlation (Cumming, 2014). * indicates *p* < .05, ** indicates *p* < .01.

**Table S19.** Correlation matrix – United States. Means, standard deviations, and correlations with confidence intervals.

| Variable | *M* | *SD* | 1 | 2 | 3 | 4 | 5 | 6 | 7 | 8 | 9 | 10 |
| --- | --- | --- | --- | --- | --- | --- | --- | --- | --- | --- | --- | --- |
|  |  |  |  |  |  |  |  |  |  |  |  |  |
| 1. Misinformation | 2.36 | 1.28 |  |  |  |  |  |  |  |  |  |  |
|  |  |  |  |  |  |  |  |  |  |  |  |  |
| 2. Age | 45.73 | 26.53 | -.15** |  |  |  |  |  |  |  |  |  |
|  |  |  | [-.22, -.07] |  |  |  |  |  |  |  |  |  |
|  |  |  |  |  |  |  |  |  |  |  |  |  |
| 3. Numeracy | 2.86 | 1.33 | -.36** | -.00 |  |  |  |  |  |  |  |  |
|  |  |  | [-.43, -.30] | [-.08, .07] |  |  |  |  |  |  |  |  |
|  |  |  |  |  |  |  |  |  |  |  |  |  |
| 4. Political ideology | 3.92 | 1.68 | .22** | .07 | -.01 |  |  |  |  |  |  |  |
|  |  |  | [.14, .28] | [-.01, .14] | [-.09, .06] |  |  |  |  |  |  |  |
|  |  |  |  |  |  |  |  |  |  |  |  |  |
| 5. Education | 3.70 | 0.90 | -.10* | .02 | .21** | -.13** |  |  |  |  |  |  |
|  |  |  | [-.17, -.02] | [-.05, .10] | [.13, .28] | [-.20, -.05] |  |  |  |  |  |  |
|  |  |  |  |  |  |  |  |  |  |  |  |  |
| 6. Compliance | 7.72 | 2.84 | -.21** | .09* | .10** | -.15** | .16** |  |  |  |  |  |
|  |  |  | [-.28, -.13] | [.02, .17] | [.03, .17] | [-.22, -.08] | [.09, .23] |  |  |  |  |  |
|  |  |  |  |  |  |  |  |  |  |  |  |  |
| 7. Trust in pols' COVID approach | 3.11 | 1.77 | .42** | -.08* | -.22** | .26** | -.03 | -.04 |  |  |  |  |
|  |  |  | [.36, .48] | [-.16, -.01] | [-.29, -.15] | [.19, .33] | [-.11, .04] | [-.11, .04] |  |  |  |  |
|  |  |  |  |  |  |  |  |  |  |  |  |  |
| 8. Trust in WHO COVID approach | 4.57 | 1.90 | -.15** | -.03 | -.04 | -.43** | .05 | .25** | .04 |  |  |  |
|  |  |  | [-.22, -.07] | [-.10, .05] | [-.11, .03] | [-.49, -.37] | [-.02, .13] | [.18, .32] | [-.03, .12] |  |  |  |
|  |  |  |  |  |  |  |  |  |  |  |  |  |
| 9. Trust in scientists | 3.87 | 0.90 | -.32** | .06 | .10** | -.35** | .19** | .26** | -.08* | .50** |  |  |
|  |  |  | [-.38, -.25] | [-.01, .13] | [.03, .18] | [-.41, -.28] | [.12, .26] | [.19, .32] | [-.15, -.01] | [.44, .55] |  |  |
|  |  |  |  |  |  |  |  |  |  |  |  |  |
| 10. Trust in journalists | 2.81 | 1.08 | -.11** | .08* | -.06 | -.35** | .13** | .19** | -.01 | .49** | .48** |  |
|  |  |  | [-.19, -.04] | [.01, .16] | [-.14, .01] | [-.41, -.28] | [.05, .20] | [.12, .26] | [-.09, .06] | [.43, .55] | [.42, .54] |  |
|  |  |  |  |  |  |  |  |  |  |  |  |  |
| 11. Trust in government | 2.58 | 1.10 | .31** | -.05 | -.13** | .33** | -.07 | -.06 | .60** | -.05 | .00 | .04 |
|  |  |  | [.25, .38] | [-.13, .02] | [-.20, -.05] | [.26, .39] | [-.14, .00] | [-.13, .01] | [.55, .64] | [-.13, .02] | [-.07, .08] | [-.04, .11] |
|  |  |  |  |  |  |  |  |  |  |  |  |  |

*Note.* *M* and *SD* are used to represent mean and standard deviation, respectively. Values in square brackets indicate the 95% confidence interval for each correlation. The confidence interval is a plausible range of population correlations that could have caused the sample correlation (Cumming, 2014). * indicates *p* < .05, ** indicates *p* < .01.

**Table S20.** Correlation matrix – Misinformation statements (all countries). Correlations with confidence intervals.

| Variable |  | 1 | 2 | 3 | 4 | 5 | 6 |
| --- | --- | --- | --- | --- | --- | --- | --- |
| 1. misinformation_5g | | — |  |  |  |  |  |
|  |  |  |  |  |  |  |  |
| 2. misinformation_breath | | 0.412*** | — |  |  |  |  |
|  |  | [0.388 - 0.434] |  |  |  |  |  |
|  |  |  |  |  |  |  |  |
| 3. misinformation_bioengineering | | 0.454*** | 0.288*** | — |  |  |  |
|  |  | [0.432 - 0.476] | [0.262 - 0.313] |  |  |  |  |
|  |  |  |  |  |  |  |  |
| 4. misinformation_hot-air | | 0.585*** | 0.497*** | 0.359*** | — |  |  |
|  |  | [0.567 - 0.603] | [0.476 - 0.518] | [0.335 - 0.383] |  |  |  |
|  |  |  |  |  |  |  |  |
| 5. misinformation_vaccination | | 0.583*** | 0.363*** | 0.549*** | 0.525*** | — |  |
|  |  | [0.564 - 0.601] | [0.338 - 0.386] | [0.529 - 0.568] | [0.505 - 0.545] |  |  |
|  |  |  |  |  |  |  |  |
| 6. misinformation_saltwater | | 0.522*** | 0.458*** | 0.334*** | 0.581*** | 0.436*** | — |
|  |  | [0.501 - 0.541] | [0.436 - 0.480] | [0.309 - 0.358] | [0.562 - 0.599] | [0.413 - 0.458] |  |
| *Note.* *** indicates *p* < 0.001. | | | | | | | |

**Table S21.** OLS for misinformation susceptibility with self-perceived minority status excluded. Significant predictors are marked in bold.

|  | *Pooled* | *Ireland* | *Mexico* | *Spain* | *UK (April)* | *USA* |
| --- | --- | --- | --- | --- | --- | --- |
| (Intercept) | 2.380 *** | 2.537 *** | 2.805 *** | 3.012 *** | 2.196 *** | 2.288 *** |
|  | [2.290, 2.471] | [2.227, 2.847] | [2.235, 3.375] | [2.687, 3.337] | [2.035, 2.356] | [2.094, 2.482] |
| Age | **-0.254 ***** | **-0.361 ***** | **0.121 *** | **-0.145 **** | **-0.362 ***** | **-0.178 ***** |
|  | [-0.288, -0.220] | [-0.457, -0.265] | [0.027, 0.214] | [-0.240, -0.050] | [-0.433, -0.291] | [-0.260, -0.096] |
| Female | **-0.165 ***** | -0.029 | -0.090 | **-0.263 **** | 0.013 | **-0.173 *** |
|  | [-0.231, -0.099] | [-0.213, 0.155] | [-0.279, 0.099] | [-0.448, -0.078] | [-0.122, 0.148] | [-0.327, -0.018] |
| Education | **0.036 *** | 0.052 | 0.030 | 0.035 | -0.049 | 0.041 |
|  | [0.002, 0.070] | [-0.040, 0.144] | [-0.065, 0.124] | [-0.057, 0.127] | [-0.117, 0.020] | [-0.040, 0.122] |
| Politically right wing | **0.089 ***** | **0.207 ***** | **0.105 *** | **0.186 ***** | 0.076 | 0.057 |
|  | [0.054, 0.123] | [0.116, 0.298] | [0.010, 0.201] | [0.083, 0.290] | [-0.001, 0.153] | [-0.040, 0.154] |
| Numeracy | **-0.408 ***** | **-0.324 ***** | **-0.328 ***** | **-0.382 ***** | **-0.370 ***** | **-0.353 ***** |
|  | [-0.441, -0.375] | [-0.416, -0.232] | [-0.424, -0.233] | [-0.474, -0.289] | [-0.438, -0.301] | [-0.435, -0.272] |
| Trust in scientists | **-0.239 ***** | **-0.287 ***** | **-0.197 ***** | **-0.229 ***** | **-0.286 ***** | **-0.294 ***** |
|  | [-0.276, -0.201] | [-0.386, -0.188] | [-0.306, -0.089] | [-0.330, -0.127] | [-0.363, -0.209] | [-0.392, -0.196] |
| Trust in government | 0.006 | 0.004 | 0.012 | -0.047 | 0.003 | **0.122 *** |
|  | [-0.038, 0.051] | [-0.109, 0.117] | [-0.113, 0.136] | [-0.176, 0.083] | [-0.091, 0.096] | [0.021, 0.224] |
| Trust in journalists | **0.125 ***** | 0.049 | **0.130 *** | **0.136 **** | **0.134 ***** | 0.035 |
|  | [0.090, 0.161] | [-0.047, 0.146] | [0.030, 0.229] | [0.035, 0.237] | [0.064, 0.205] | [-0.062, 0.131] |
| COVID risk perception | -0.024 | -0.055 | -0.057 | -0.058 | 0.050 | -0.029 |
|  | [-0.058, 0.010] | [-0.147, 0.036] | [-0.156, 0.041] | [-0.154, 0.039] | [-0.020, 0.120] | [-0.117, 0.059] |
| Trust in pols' COVID approach | **0.135 ***** | -0.037 | **0.270 ***** | **0.155 *** | 0.082 | **0.329 ***** |
|  | [0.090, 0.180] | [-0.153, 0.080] | [0.149, 0.391] | [0.031, 0.278] | [-0.010, 0.175] | [0.228, 0.429] |
| Trust in WHO's COVID appr. | **-0.096 ***** | -0.023 | **-0.281 ***** | 0.055 | -0.028 | -0.059 |
|  | [-0.135, -0.056] | [-0.132, 0.087] | [-0.392, -0.170] | [-0.052, 0.162] | [-0.107, 0.052] | [-0.163, 0.044] |
| Getting info from social media | **0.327 ***** | **0.289 **** | 0.294 | 0.182 | **0.253 **** | **0.268 **** |
|  | [0.248, 0.406] | [0.084, 0.493] | [-0.178, 0.766] | [-0.080, 0.443] | [0.099, 0.407] | [0.091, 0.445] |
| Getting info from WHO | **-0.108 *** | -0.298 | -0.289 | **-0.416 *** | -0.094 | -0.040 |
|  | [-0.199, -0.018] | [-0.602, 0.006] | [-0.699, 0.121] | [-0.737, -0.095] | [-0.257, 0.070] | [-0.237, 0.156] |
| N | 4904 | 676 | 689 | 686 | 1035 | 693 |
| R^2^ | 0.228 | 0.269 | 0.198 | 0.197 | 0.271 | 0.364 |
| *** *p* < 0.001; ** *p* < 0.01; * *p* < 0.05. | | | | | | |

**Table S22.** Logistic regression model for vaccine hesitancy, by country, with minority status excluded.

|  |  | **Would you get vaccinated against COVID yourself** | | | | | | | **Would you recommend others to get vaccinated** | | | | | | |
| --- | --- | --- | --- | --- | --- | --- | --- | --- | --- | --- | --- | --- | --- | --- | --- |
| **Country** | **Variable** | *Estimate* | *SE* | *z* | *p* | *OR* | *2.50%* | *97.50%* | *Estimate* | *SE* | *z* | *p* | *OR* | *2.50%* | *97.50%* |
| *Pooled* | (Intercept) | -0.287 | 0.328 | -0.875 | 0.381 | 0.751 | 0.395 | 1.426 | 0.016 | 0.348 | 0.047 | 0.963 | 1.016 | 0.514 | 2.011 |
|  | Age | 0.009 | 0.003 | 3.171 | 0.002 | **1.009** | 1.004 | 1.015 | 0.008 | 0.003 | 2.415 | 0.016 | **1.008** | 1.001 | 1.014 |
|  | Female | -0.384 | 0.09 | -4.249 | 0 | **0.681** | 0.57 | 0.813 | -0.303 | 0.097 | -3.105 | 0.002 | **0.739** | 0.61 | 0.894 |
|  | Education | 0.019 | 0.041 | 0.458 | 0.647 | 1.019 | 0.94 | 1.104 | 0.003 | 0.044 | 0.06 | 0.952 | 1.003 | 0.919 | 1.093 |
|  | Politically right wing | -0.045 | 0.031 | -1.428 | 0.153 | 0.956 | 0.899 | 1.017 | -0.019 | 0.034 | -0.569 | 0.569 | 0.981 | 0.918 | 1.048 |
|  | Numeracy | 0.005 | 0.036 | 0.139 | 0.89 | 1.005 | 0.937 | 1.078 | 0.004 | 0.039 | 0.104 | 0.918 | 1.004 | 0.931 | 1.083 |
|  | Trust in scientists | 0.548 | 0.048 | 11.525 | 0 | **1.73** | 1.577 | 1.901 | 0.587 | 0.05 | 11.654 | 0 | **1.799** | 1.631 | 1.987 |
|  | Misinformation | -0.259 | 0.035 | -7.362 | 0 | **0.772** | 0.72 | 0.827 | -0.326 | 0.037 | -8.719 | 0 | **0.722** | 0.671 | 0.777 |
| *Mexico* | (Intercept) | -0.041 | 0.671 | -0.061 | 0.951 | 0.96 | 0.258 | 3.599 | 0.051 | 0.684 | 0.074 | 0.941 | 1.052 | 0.276 | 4.05 |
|  | Age | -0.008 | 0.006 | -1.194 | 0.232 | 0.992 | 0.98 | 1.005 | -0.009 | 0.006 | -1.391 | 0.164 | 0.991 | 0.979 | 1.004 |
|  | Female | -0.736 | 0.189 | -3.89 | 0 | **0.479** | 0.329 | 0.692 | -0.528 | 0.192 | -2.753 | 0.006 | **0.59** | 0.403 | 0.857 |
|  | Education | 0.013 | 0.105 | 0.124 | 0.901 | 1.013 | 0.822 | 1.242 | 0.027 | 0.107 | 0.256 | 0.798 | 1.028 | 0.831 | 1.264 |
|  | Politically right wing | 0.162 | 0.071 | 2.284 | 0.022 | **1.176** | 1.024 | 1.352 | 0.109 | 0.072 | 1.508 | 0.131 | 1.115 | 0.968 | 1.286 |
|  | Numeracy | -0.022 | 0.078 | -0.287 | 0.774 | 0.978 | 0.839 | 1.138 | -0.012 | 0.08 | -0.147 | 0.883 | 0.988 | 0.845 | 1.155 |
|  | Trust in scientists | 0.494 | 0.096 | 5.117 | 0 | **1.638** | 1.358 | 1.984 | 0.526 | 0.098 | 5.382 | 0 | **1.692** | 1.4 | 2.055 |
|  | Misinformation | -0.259 | 0.071 | -3.658 | 0 | **0.772** | 0.671 | 0.886 | -0.288 | 0.072 | -4.018 | 0 | **0.75** | 0.651 | 0.862 |
| *Spain* | (Intercept) | -0.735 | 0.743 | -0.99 | 0.322 | 0.479 | 0.111 | 2.053 | -0.029 | 0.776 | -0.038 | 0.97 | 0.971 | 0.212 | 4.468 |
|  | Age | 0.003 | 0.007 | 0.44 | 0.66 | 1.003 | 0.99 | 1.016 | 0.004 | 0.007 | 0.605 | 0.545 | 1.004 | 0.99 | 1.019 |
|  | Female | -0.479 | 0.202 | -2.375 | 0.018 | **0.619** | 0.416 | 0.917 | -0.357 | 0.213 | -1.679 | 0.093 | 0.699 | 0.459 | 1.06 |
|  | Education | 0.071 | 0.092 | 0.767 | 0.443 | 1.073 | 0.896 | 1.285 | -0.051 | 0.098 | -0.527 | 0.598 | 0.95 | 0.784 | 1.149 |
|  | Politically right wing | -0.026 | 0.067 | -0.386 | 0.7 | 0.974 | 0.855 | 1.112 | -0.065 | 0.071 | -0.928 | 0.353 | 0.937 | 0.816 | 1.076 |
|  | Numeracy | 0.016 | 0.083 | 0.186 | 0.852 | 1.016 | 0.862 | 1.196 | 0.048 | 0.088 | 0.542 | 0.588 | 1.049 | 0.882 | 1.246 |
|  | Trust in scientists | 0.582 | 0.106 | 5.47 | 0 | **1.789** | 1.456 | 2.211 | 0.581 | 0.111 | 5.257 | 0 | **1.788** | 1.442 | 2.227 |
|  | Misinformation | -0.086 | 0.08 | -1.073 | 0.283 | 0.917 | 0.785 | 1.075 | -0.133 | 0.083 | -1.592 | 0.111 | 0.876 | 0.744 | 1.033 |
| *UK* | (Intercept) | -0.589 | 0.581 | -1.014 | 0.311 | 0.555 | 0.177 | 1.73 | -0.568 | 0.64 | -0.887 | 0.375 | 0.567 | 0.161 | 1.984 |
|  | Age | 0.021 | 0.006 | 3.719 | 0 | **1.021** | 1.01 | 1.032 | 0.014 | 0.006 | 2.296 | 0.022 | **1.014** | 1.002 | 1.027 |
|  | Female | -0.167 | 0.159 | -1.045 | 0.296 | 0.847 | 0.619 | 1.156 | -0.075 | 0.18 | -0.418 | 0.676 | 0.928 | 0.651 | 1.319 |
|  | Education | 0.038 | 0.064 | 0.587 | 0.557 | 1.038 | 0.916 | 1.178 | 0.036 | 0.072 | 0.497 | 0.619 | 1.036 | 0.9 | 1.194 |
|  | Politically right wing | -0.03 | 0.06 | -0.495 | 0.62 | 0.971 | 0.864 | 1.091 | 0.07 | 0.067 | 1.031 | 0.303 | 1.072 | 0.939 | 1.224 |
|  | Numeracy | 0.025 | 0.061 | 0.406 | 0.685 | 1.025 | 0.909 | 1.154 | -0.023 | 0.068 | -0.342 | 0.732 | 0.977 | 0.854 | 1.116 |
|  | Trust in scientists | 0.482 | 0.083 | 5.801 | 0 | **1.619** | 1.377 | 1.908 | 0.633 | 0.092 | 6.876 | 0 | **1.884** | 1.575 | 2.261 |
|  | Misinformation | -0.288 | 0.063 | -4.561 | 0 | **0.75** | 0.662 | 0.848 | -0.357 | 0.07 | -5.074 | 0 | **0.7** | 0.609 | 0.803 |
| *USA* | (Intercept) | -1.038 | 0.775 | -1.339 | 0.181 | 0.354 | 0.076 | 1.606 | -0.883 | 0.837 | -1.055 | 0.292 | 0.414 | 0.079 | 2.12 |
|  | Age | 0.012 | 0.006 | 1.921 | 0.055 | 1.012 | 1 | 1.025 | 0.012 | 0.007 | 1.721 | 0.085 | 1.012 | 0.998 | 1.027 |
|  | Female | -0.306 | 0.194 | -1.576 | 0.115 | 0.736 | 0.502 | 1.076 | -0.395 | 0.215 | -1.84 | 0.066 | 0.674 | 0.441 | 1.024 |
|  | Education | 0.143 | 0.112 | 1.276 | 0.202 | 1.153 | 0.926 | 1.438 | 0.312 | 0.123 | 2.533 | 0.011 | **1.366** | 1.074 | 1.742 |
|  | Politically right wing | -0.223 | 0.065 | -3.418 | 0.001 | **0.8** | 0.703 | 0.908 | -0.19 | 0.072 | -2.645 | 0.008 | **0.827** | 0.718 | 0.951 |
|  | Numeracy | 0.026 | 0.079 | 0.331 | 0.741 | 1.026 | 0.879 | 1.198 | -0.027 | 0.086 | -0.316 | 0.752 | 0.973 | 0.82 | 1.152 |
|  | Trust in scientists | 0.714 | 0.116 | 6.169 | 0 | **2.042** | 1.633 | 2.573 | 0.735 | 0.124 | 5.909 | 0 | **2.086** | 1.641 | 2.675 |
|  | Misinformation | -0.228 | 0.081 | -2.794 | 0.005 | **0.796** | 0.678 | 0.935 | -0.372 | 0.088 | -4.227 | 0 | **0.689** | 0.579 | 0.818 |

**Table S23.** OLS for compliance with health guidance, with self-perceived minority status excluded. Significant predictors are marked in bold.

|  | *Pooled* | *Ireland* | *Mexico* | *Spain* | *UK (April)* | *United States* |
| --- | --- | --- | --- | --- | --- | --- |
| (Intercept) | 7.106 *** | 7.106 *** | 8.327 *** | 7.559 *** | 6.604 *** | 7.261 *** |
|  | [7.006, 7.205] | [6.859, 7.353] | [8.086, 8.567] | [7.279, 7.838] | [6.396, 6.812] | [6.996, 7.525] |
| Age | **0.175 ***** | **0.294 **** | **0.478 ***** | 0.021 | 0.119 | **0.365 ***** |
|  | [0.102, 0.248] | [0.104, 0.483] | [0.308, 0.648] | [-0.179, 0.222] | [-0.038, 0.276] | [0.171, 0.559] |
| Female | **0.687 ***** | **0.819 ***** | **0.587 ***** | **0.464 *** | **0.708 ***** | **0.924 ***** |
|  | [0.547, 0.828] | [0.461, 1.177] | [0.247, 0.928] | [0.066, 0.862] | [0.418, 0.998] | [0.551, 1.297] |
| Education | **0.263 ***** | -0.016 | 0.027 | 0.063 | **0.192 *** | **0.227 *** |
|  | [0.192, 0.335] | [-0.197, 0.165] | [-0.144, 0.197] | [-0.135, 0.262] | [0.044, 0.341] | [0.035, 0.420] |
| Politically right wing | 0.035 | -0.065 | 0.004 | **0.300 **** | **0.198 **** | -0.015 |
|  | [-0.036, 0.106] | [-0.241, 0.112] | [-0.166, 0.173] | [0.097, 0.503] | [0.051, 0.344] | [-0.225, 0.195] |
| Numeracy | 0.075 | **0.212 *** | 0.051 | -0.009 | 0.148 | 0.156 |
|  | [-0.000, 0.150] | [0.024, 0.399] | [-0.127, 0.229] | [-0.218, 0.200] | [-0.009, 0.305] | [-0.048, 0.361] |
| COVID risk perception | **0.765 ***** | **0.611 ***** | **0.688 ***** | **0.599 ***** | **0.839 ***** | **0.981 ***** |
|  | [0.693, 0.837] | [0.433, 0.789] | [0.515, 0.860] | [0.394, 0.803] | [0.690, 0.987] | [0.779, 1.184] |
| Trust in scientists | **0.271 ***** | **0.043** | **0.198 *** | **0.235 *** | 0.055 | **0.256 *** |
|  | [0.197, 0.344] | [-0.138, 0.224] | [0.022, 0.374] | [0.029, 0.441] | [-0.098, 0.208] | [0.042, 0.470] |
| Misinformation | **-0.127 **** | -0.030 | **-0.342 ***** | **-0.303 **** | -0.007 | -0.185 |
|  | [-0.205, -0.050] | [-0.229, 0.169] | [-0.522, -0.162] | [-0.518, -0.088] | [-0.172, 0.158] | [-0.401, 0.030] |
| N | 4918 | 677 | 690 | 687 | 1037 | 693 |
| R^2^ | 0.155 | 0.133 | 0.177 | 0.108 | 0.164 | 0.246 |
| *** *p* < 0.001; ** *p* < 0.01; * *p* < 0.05. | | | | | | |

**Table S24.** OLS for susceptibility to misinformation, with the question “would you get vaccinated against COVID-19” included. Significant predictors are marked in bold.

|  | *Pooled* | *Mexico* | *Spain* | *UK (May)* | *United States* |
| --- | --- | --- | --- | --- | --- |
| (Intercept) | 2.837 *** | 2.999 *** | 2.830 *** | 2.737 *** | 2.595 *** |
|  | [2.730, 2.944] | [2.750, 3.248] | [2.583, 3.078] | [2.570, 2.904] | [2.381, 2.809] |
| Age | **-0.196 ***** | **0.107 *** | **-0.150 **** | **-0.335 ***** | **-0.241 ***** |
|  | [-0.239, -0.153] | [0.008, 0.207] | [-0.246, -0.055] | [-0.404, -0.267] | [-0.327, -0.155] |
| Female | **-0.246 ***** | -0.089 | **-0.245 *** | **-0.340 ***** | **-0.178 *** |
|  | [-0.331, -0.162] | [-0.291, 0.113] | [-0.435, -0.055] | [-0.470, -0.210] | [-0.346, -0.010] |
| Education | **0.047 *** | 0.022 | 0.032 | -0.026 | 0.046 |
|  | [0.004, 0.090] | [-0.079, 0.122] | [-0.063, 0.127] | [-0.093, 0.041] | [-0.041, 0.132] |
| Politically right wing | **0.121 ***** | **0.101 *** | **0.157 **** | **0.087 **** | **0.233 ***** |
|  | [0.078, 0.163] | [0.001, 0.201] | [0.061, 0.253] | [0.021, 0.153] | [0.138, 0.328] |
| Self-perceived minority status | **0.383 ***** | **0.395 **** | **0.357 **** | **0.516 ***** | **0.276 **** |
|  | [0.275, 0.491] | [0.154, 0.635] | [0.100, 0.613] | [0.341, 0.692] | [0.071, 0.481] |
| Numeracy | **-0.431 ***** | **-0.333 ***** | **-0.390 ***** | **-0.418 ***** | **-0.424 ***** |
|  | [-0.474, -0.388] | [-0.435, -0.232] | [-0.485, -0.294] | [-0.485, -0.351] | [-0.509, -0.338] |
| COVID risk perception | -0.005 | -0.061 | -0.055 | 0.043 | -0.003 |
|  | [-0.049, 0.039] | [-0.164, 0.043] | [-0.153, 0.043] | [-0.025, 0.111] | [-0.099, 0.093] |
| Trust in scientists | **-0.159 ***** | **-0.184 ***** | **-0.170 ***** | **-0.142 ***** | **-0.244 ***** |
|  | [-0.204, -0.115] | [-0.288, -0.080] | [-0.269, -0.070] | [-0.210, -0.074] | [-0.340, -0.148] |
| Would get COVID vaccine | **-0.391 ***** | **-0.359 **** | -0.158 | **-0.444 ***** | **-0.286 **** |
|  | [-0.497, -0.284] | [-0.601, -0.116] | [-0.404, 0.087] | [-0.612, -0.276] | [-0.503, -0.070] |
| N | 3091 | 645 | 664 | 1109 | 673 |
| R^2^ | 0.216 | 0.149 | 0.179 | 0.294 | 0.280 |
| *** *p* < 0.001; ** *p* < 0.01; * *p* < 0.05. | | | | | |

**Table S25.** Measurement invariance test using MG-CFA for the 6 misinformation statements.

| **Chi-squared difference test, with "country" as group variable** | | | | |  |  |  |
| --- | --- | --- | --- | --- | --- | --- | --- |
|  | *Df* | *AIC* | *BIC* | *χ^2^* | *Δχ^2^* | *ΔDf* | *p* |
| fit.configural | 54 | 108419 | 109123 | 812.4 |  |  |  |
| fit.loadings (weak) | 79 | 108422 | 108962 | 864.67 | 52.271 | 25 | 0.001107** |
| fit.intercepts (strong) | 104 | 108602 | 108980 | 1095.32 | 230.645 | 25 | <2.2e-16*** |
| fit.means (strict) | 109 | 108678 | 109023 | 1180.67 | 85.35 | 5 | <2.2e-16*** |
|  |  |  |  |  |  |  |  |
| **Fit measures** |  |  |  |  |  |  |  |
|  | *CFI* | *RMSEA* | *ΔCFI* | *ΔRMSEA* |  |  |  |
| fit.configural | 0.931 | 0.13 |  |  |  |  |  |
| fit.loadings (weak) | 0.929 | 0.109 | 0.002 | 0.021 |  |  |  |
| fit.intercepts (strong) | 0.91 | 0.107 | 0.019 | 0.002 |  |  |  |
| fit.means (strict) | 0.903 | 0.109 | 0.007 | 0.002 |  |  |  |
| *** *p* < 0.001; ** *p* < 0.01; * *p* < 0.05. | | |  |  |  |  |  |

1. For the “Vaccination-self” and “Vaccination-recommend” questions, the large number of missing data points is explained by the fact that these questions were not asked in the Irish and first UK surveys (these were only added to the surveys that were conducted later). [↑](#footnote-ref-1)
